# SV-BR-1-GM, a Clinically Effective GM-CSF-Secreting Breast Cancer Cell Line, Expresses an Immune Signature and Directly Activates CD4^+^ T Lymphocytes

**DOI:** 10.3389/fimmu.2018.00776

**Published:** 2018-05-15

**Authors:** Markus D. Lacher, Gerhard Bauer, Brian Fury, Sanne Graeve, Emily L. Fledderman, Tye D. Petrie, Dane P. Coleal-Bergum, Tia Hackett, Nicholas H. Perotti, Ying Y. Kong, William W. Kwok, Joseph P. Wagner, Charles L. Wiseman, William V. Williams

**Affiliations:** ^1^BriaCell Therapeutics Corp., Berkeley, CA, United States; ^2^GMP Facility, Institute for Regenerative Cures, University of California, Davis (UCD), Sacramento, CA, United States; ^3^Benaroya Research Institute at Virginia Mason, Seattle, WA, United States

**Keywords:** SV-BR-1-GM, GVAX, targeted immunotherapy, whole-cell vaccine, therapeutic cancer vaccine, antigen-presenting cells

## Abstract

Targeted cancer immunotherapy with irradiated, granulocyte–macrophage colony-stimulating factor (GM-CSF)-secreting, allogeneic cancer cell lines has been an effective approach to reduce tumor burden in several patients. It is generally assumed that to be effective, these cell lines need to express immunogenic antigens coexpressed in patient tumor cells, and antigen-presenting cells need to take up such antigens then present them to patient T cells. We have previously reported that, in a phase I pilot study (ClinicalTrials.gov NCT00095862), a subject with stage IV breast cancer experienced substantial regression of breast, lung, and brain lesions following inoculation with clinical formulations of SV-BR-1-GM, a GM-CSF-secreting breast tumor cell line. To identify diagnostic features permitting the prospective identification of patients likely to benefit from SV-BR-1-GM, we conducted a molecular analysis of the SV-BR-1-GM cell line and of patient-derived blood, as well as a tumor specimen. Compared to normal human breast cells, SV-BR-1-GM cells overexpress genes encoding tumor-associated antigens (TAAs) such as PRAME, a cancer/testis antigen. Curiously, despite its presumptive breast epithelial origin, the cell line expresses major histocompatibility complex (MHC) class II genes (*HLA-DRA, HLA-DRB3, HLA-DMA, HLA-DMB*), in addition to several other factors known to play immunostimulatory roles. These factors include MHC class I components (*B2M, HLA-A, HLA-B*), *ADA* (encoding adenosine deaminase), *ADGRE5* (*CD97*), *CD58* (*LFA3*), *CD74* (encoding invariant chain and CLIP), *CD83, CXCL8* (*IL8*), *CXCL16, HLA-F, IL6, IL18*, and *KITLG*. Moreover, both SV-BR-1-GM cells and the responding study subject carried an HLA-DRB3*02:02 allele, raising the question of whether SV-BR-1-GM cells can directly present endogenous antigens to T cells, thereby inducing a tumor-directed immune response. In support of this, SV-BR-1-GM cells (which also carry the HLA-DRB3*01:01 allele) treated with yellow fever virus (YFV) envelope (Env) 43–59 peptides reactivated YFV-DRB3*01:01-specific CD4^+^ T cells. Thus, the partial HLA allele match between SV-BR-1-GM and the clinical responder might have enabled patient T lymphocytes to directly recognize SV-BR-1-GM TAAs as presented on SV-BR-1-GM MHCs. Taken together, our findings are consistent with a potentially unique mechanism of action by which SV-BR-1-GM cells can act as APCs for previously primed CD4^+^ T cells.

## Introduction

In contrast to traditional chemo- or radiation therapies that kill fast-dividing cells irrespective of whether they are cancerous or normal, the goal of cancer immunotherapy is to eliminate malignant cells based on their antigenic makeup, their tumor-associated antigens (TAAs). There are several viable ways to induce an immune response against TAAs, in part determined by whether the antigens are localized intra- or extracellularly. For instance, although chimeric antigen receptors (1, 2) and bispecific antibodies crosslinking cytotoxic T cells with cancer cells (3) rely on the antigens’ cell surface presence, ectopic T cell receptor (4), tumor-infiltrating lymphocyte (TIL) (5, 6), and vaccine-based approaches (7–18) require display of antigenic peptides by major histocompatibility complexes (MHCs), regardless of whether the peptides represent intra- or extracellular TAAs.

Whole-cell preparations with live but irradiated cancer cells express a very large number of antigens of which some may be coexpressed in the patient’s tumor(s) (19). Although a tumor shielded by an immune-suppressive microenvironment may not elicit an immune response, whole-cell preparations, if injected into immune-permissive sites, may allow development of otherwise inhibited antibody and cell-mediated immunity. However, even though an immune response induced by the injected cells may have a tumor-directed component, and even elicit tumor regression, the antigen(s) mediating this effect rarely are known.

For targeted immunotherapy studies with whole-cell preparations (also referred to as therapeutic cancer vaccines), both autologous and allogeneic cells have been applied. Autologous cancer cells, derived from the tumor of the patient to be treated, almost, per definition, are expected to express relevant antigens, including patient-specific neoepitopes. On the other hand, while allogeneic cell lines engineered to express granulocyte–macrophage colony-stimulating factor (GM-CSF) may induce strong immune responses by promoting antigen display on dendritic cells (DCs), they may lack key antigens (9, 15). With variable success, therapeutic cancer vaccines have been clinically tested against a variety of malignancies representing both hematologic and solid cancers, such as leukemia, melanoma, pancreatic, prostate, breast, lung, and colon cancers (7, 8, 10, 11, 14, 16, 18, 20–23). Notably, in a mouse model, Ogawa et al. demonstrated that a similar approach may also be suitable to *prevent* tumor establishment (prophylactic treatment), i.e., that whole-cell preparations may prevent the development of tumors and not solely serve to reduce the tumor burden of already existing disease (24).

We previously established a cell line from a chest wall lesion of a metastatic breast cancer patient (17). The cell line, referred to as SV-BR-1, is estrogen receptor/progesterone receptor negative and very strongly HER2/neu (ERBB2) positive (17). To enhance the cells’ immune reactivity, SV-BR-1 cells were genetically engineered to stably overexpress GM-CSF, yielding the SV-BR-1-*GM* cell line. Several advanced-stage cancer patients, mostly with breast cancer, were treated with irradiated (200 Gy) SV-BR-1-GM cells (16). The study employed a pretreatment step with low-dose cyclophosphamide, which has similarly been used in other studies to blunt the activity of regulatory T cells (25). Additionally, 2 and 4 days after the administration of the SV-BR-1-GM cells, interferon-alpha 2b (IFN-α2b) was injected into each inoculation site to provide an additional “danger signal” (16, 26).

In our initial round of clinical assessment, four evaluable patients completed the SV-BR-1-GM program. One subject responded to the regimen with a near-complete regression of multiple breast lesions and a complete remission of a lung metastasis but relapsed 3 months after the sixth and last cycle, with lesions in the lung, soft tissue, breast, and brain. After obtaining Food and Drug Administration (FDA) permission, treatment resumed. Consequently, a systemic response was observed whereby tumors at multiple sites, including the brain, promptly regressed (16).

Here, we describe a molecular fingerprint of SV-BR-1-GM established with samples representing developmental intermediates including master cell banks (MCBs) and drug product. We present several lines of evidence suggesting that SV-BR-1-GM cells can act as antigen-presenting cells (APCs) and thereby mount an effective tumor-directed immune response. In particular, while likely other aspects such as cross-presentation by DCs contribute to SV-BR-1-GM’s mechanism of action (MoA), our observations are consistent with a role in which SV-BR-1-GM cells express, process, and display TAAs directly to T cells. However, SV-BR-1-GM cells do not express *CD80* or *CD86*, encoding ligands for the costimulatory receptor CD28, and are thus likely to only activate previously primed, rather than naïve, T cells.

## Materials and Methods

### Culturing of Cells

SV-BR-1-GM lots were manufactured in RPMI-1640 supplemented with 10% FBS and l-glutamine or Gibco GlutaMAX (Thermo Fisher Scientific, Waltham, MA, USA) (full medium). Typically, for culture expansion toward cell banks, media changes were conducted with only about 50% of new medium. For early lots, SV-BR-1-GM cells were expanded from cryopreserved cell suspensions starting from T-25 flasks with sequential propagation in larger flasks and harvesting from about thirty T-150 flasks. Current lots are expanded in 10-STACK CellSTACK Culture Chambers (Corning Inc., Corning, NY, USA). A549 cells were obtained from the American Type Culture Collection (ATCC; Manassas, VA, USA) and cultured in ATCC-formulated F-12K medium supplemented with 10% fetal bovine serum.

### Microarray Gene Expression Profiling

SV-BR-1-GM cells, obtained directly from cryogenic vials following recovery from liquid nitrogen storage or harvested from cultures, were lysed in Buffer RLT (Qiagen, Valencia, CA, USA) with or without supplementation with β-mercaptoethanol. Total RNA was isolated from lysates *via* RNeasy Mini Kits (Qiagen) then subjected to microarray hybridization at the University of Minnesota Genomics Center (MN, USA). In short, RNA was amplified as antisense RNA and biotinylated using the Illumina™ TotalPrep™-96 RNA Amplification Kit (Thermo Fisher Scientific) according to the manufacturer’s instructions. The biotinylated antisense RNA was then hybridized onto HumanHT-12 v4 Expression BeadChip arrays (Illumina, San Diego, CA, USA) and thereafter stained with Cy3-streptavidin. Fluorescent signal intensities were acquired on an iScan array scanner (Illumina). Average signal intensities and detection *p*-values were calculated using *GenomeStudio* (Illumina). Thereafter, non-normalized data sets passing below defined quality control (QC) criteria were analyzed with various modules of *GenePattern* using the public server portal (http://www.broadinstitute.org/cancer/software/genepattern/) (27). If applicable, datasets to be compared were merged using the *MergeColumns* version 1 module. Expression levels of all Illumina samples to be cross-compared were quantile-normalized using the *IlluminaNormalizer* version 2 (beta) module then further processed in *Microsoft Excel* and/or subjected to log transformation and hierarchical clustering *via* the *HierarchicalClustering* version 6 module (distance correlation: *Pearson correlation*; clustering method: *Pairwise average-linkage*). Heat maps and dendrograms of clustered data were generated using the *HierarchicalClusteringViewer* version 11 module. To compare gene expression levels between SV-BR-1-GM and samples analyzed by others, Gene Expression Omnibus [(GEO); National Center for Biotechnology Information (NCBI)] (28) DataSets, also generated on the Illumina HumanHT-12 v4 Expression BeadChip platform, were merged with SV-BR-1-GM data sets and processed as described above. For the *in silico* analyses of GEO DataSets generated on Affymetrix Human Genome U133 Plus 2.0 Arrays, CEL files were RMA/quantile-normalized and background-subtracted using the *ExpressionFileCreator* module of *GenePattern* then filtered in Microsoft Excel as described subsequently.

A gene was defined as expressed if at least one corresponding probe yielded a quantile-normalized expression value above the median “expression” level among all human RNA-targeting, non-control, probes (max. 47323 for the HumanHT-12 v4 Expression BeadChip arrays, Illumina). This background cutoff definition coincides with the roughly 50% of genes expressed in a collection of human tissues at levels detectable by massively parallel signature sequencing in a study by Jongeneel et al. (29). However, since the tissues analyzed must have contained an unknown number of different cell types and unknown relative contributions of each cell type to the overall number of cells, this definition likely overestimates the extent of actual background. Nevertheless, consequently, it may reduce the probability of calling nonexpressed genes expressed.

### QC of Non-Normalized Data Sets

The integrity of preamplified SV-BR-1-GM RNA was determined *via* Agilent’s 4200 TapeStation system (Agilent, Santa Clara, CA, USA). Samples with an RNA integrity number equivalent (RIN^e^) value of less than 7.5 were excluded from further analyses. Additionally, for SV-BR-1-GM as well as samples obtained *via* GEO (NCBI) and processed on HumanHT-12 v.4 BeadChips, non-normalized data sets were assessed for gene expression variability. Except where stated otherwise, low-variability samples were excluded from further processing, with low-variability defined as a ratio between the expression value at the 95th and 5th, respectively, percentile of less than 10.

### Sample Representation

For comparative gene expression analyses, individual genes were represented in the various SV-BR-1-GM sample types [MCB cryo, clinical product (CP) Lot IV culture, CP Lot IV 4p cryo, CP Lot IV 4p culture, CP Lot V cryo, CP Lot VIII cryo, CP Lot VIII culture 1d, CP Lot VIII culture 3d, and RES Lot II cryo] by their arithmetic means of their gene expression values. For calculations requiring one representative SV-BR-1-GM gene expression value, the median value among the arithmetic means was used. Representative gene expression levels for samples other than SV-BR-1-GM, obtained from GEO, were defined as follows: Breast cancer cell line samples from DataSet GSE48398 and human mammary epithelial cell samples (HMECs, “early proliferating” vs. “deep senescence”, treated with siGLO siRNA) from DataSet GSE56718 (30) were represented by their arithmetic means. Normal breast sample types (ALDH NEG, ALDH POS, ERBB3 NEG, NCL, BASAL, STROMAL) from DataSet GSE35399 (31) were represented by their median expression values unless stated otherwise. For DataSet GSE48398, only expression profiles from cells cultured at 37°C were utilized. For the comparison between the breast cancer (DataSet GSE2943) and the normal tissues (DataSet GSE7307), the 95th percentile values among all breast cancer tissues (HER2_3^+^, HER2_2^+^, HER2_0-1^+^ of GSE2943) and the 95th percentile values among the *maximum* expression values of each group of normal tissue (Data Sheet S1 in Supplementary Material, GSE7307) were used as comparators. The 95th percentile rather than maximum (of the max.) expression values were chosen to accommodate potential “outliers.” Cancer/testis antigen (CTA) overexpression in SV-BR-1-GM cells was defined by the following criteria: The representative CTA transcript level in SV-BR-1-GM cells was to be both >1.5 times the background cutoff value AND >1.5 times the max. transcript level among the non-cultured (NC) normal breast cell types (SV-BR-1-GM/Max), AND the max. CTA transcript level among the NC normal breast cell types was to be <1.5 times the background cutoff value, whereby the max. NC transcript level was established among the representative values of each sample type.

### *In Silico* Identification of Putative SV-BR-1-GM TAAs

Quantile-normalized SV-BR-1-GM gene expression values were compared to those of normal human breast cells represented by the GEO DataSets GSE35399 (31), GSE56718 (30), and MCF10A from GSE48398. Genes for which the representative SV-BR-1-GM expression value was both >1.5 times the background cutoff value (defined above) and >1.5 times higher than the maximum representative value among all groups of normal breast cells were additionally subjected to the second, medium stringency, filtration step (expression level > 5 times the background cutoff value). Verification of the genes retained after medium stringency filtration was done *via* the quotients of the representative breast cancer samples in GSE2943 and those of the quantile-normalized and grouped normal tissues in GEO DataSet GSE7307 (high stringency filter). Groups of normal tissues are listed in Data Sheet S1 in Supplementary Material. As cutoff value for high stringency filter retention served the quotient (Breast Cancer/Normal Tissues) value for the *ERBB2* Affymetrix probe 216836_s_at (quotient = 3.95): Genes of probes for which the quotient is ≥3.00 were defined as “verified.”

To assess transcript expression levels of 279 confirmed or putative CTAs (Data Sheets S4 in Supplemental Material) in SV-BR-1-GM cells in comparison to several other breast cancer cell lines and normal breast cells, GEO DataSets of both cultured [GSE56718 (30) and GSE48398 (MCF10A)] and noncultured [GSE35399 (31)] normal breast cells were utilized. The CTA genes chosen for the analysis were selected from those described by Dobrynin et al., (2013) (32) and Chapman et al. (33), those listed in the CT database (34), and those represented by the nCounter Human PanCancer Immune Profiling Panel (NanoString Technologies, Seattle, WA, USA).

### Verification of Expression of Immune-Related Genes by Quantitative Reverse Transcription-Polymerase Chain Reaction (qRT-PCR), Transcript Counting (nCounter), ELISA, and Flow Cytometry

To confirm expression of immune-related genes, several methods were employed. For a subset of the genes, expression was assessed by qRT-PCR at the University of Minnesota Genomics Center (MN, USA) using commercially available TaqMan assays (Table S1 in Supplementary Data Sheet S2 in Supplementary Material) and the (nonirradiated) samples listed in Table S2 in Supplementary Data Sheet S2 in Supplementary Material, which were also assessed by microarray hybridization. Data were acquired on an ABI 7900HT real-time PCR instrument. To establish whether the immune-related genes are also expressed by irradiated SV-BR-1-GM cells from clinical formulations, cells were irradiated with 200 Gy then resuspended in Lactated Ringer’s solution (LRS) and courier-transported under temperature controlled (2–8°C) conditions from the manufacturing site (UC Davis GMP facility, Sacramento, CA, USA) to the processing laboratory in Berkeley, CA, USA. The shipping containers (Crēdo Cube™ Series 4 parcel shippers; Pelican BioThermal, Plymouth, MN, USA) were opened, 4 h and 24 h after completion of the formulation process. Samples were immediately thereafter assessed for cell viability, seeded in 6-well plates, and cultured in full medium. Supernatants (SNs) and cells were harvested from the original clinical formulations, and after 1 and 3 days of culturing. RNA (extracted *via* RNeasy Mini Kit; Qiagen) was subjected to nCounter-based transcript counting (NanoString Technologies) at the University of Minnesota Genomics Center with the CodeSets (probes) listed in Table S3 in Supplementary Data Sheet S2 in Supplementary Material. Using nSolver Version 4.0 (NanoString), data was background-subtracted and normalized against the system positive controls and the reference genes (*ADRM1, APTX, DGUOK, GNG5, PSMA4, RPL38, TMEM14C*, and *UBE3C*), using the geometric means for both the positive control and reference gene sets. For the background subtraction, the max. values of the system negative controls were subtracted.

To assess protein expression levels of key immune-related factors, SNs from samples derived from the 4- and 24-h-old clinical formulations were subjected to ELISA using the Human GM-CSF, IL-6, IL-8, IL-10, IL-15, and KITLG (free SCF) ELISA MAX™ Deluxe kits (BioLegend, Inc., San Diego, CA, USA).

To establish whether and to what extent human leukocyte antigen (HLA)-DRβ3 (encoded by *HLA-DRB3*) is expressed on the surface of irradiated SV-BR-1-GM cells, cells were subjected to a modified formulation process whereby cells, after harvesting using TrypLE Express (Thermo Fisher Scientific) and irradiation (200 Gy), were cryopreserved in CryoStor 5 (CS5) cell freeze medium (BioLife Solutions, Inc., Bothell, WA, USA) then shipped overnight, on dry ice, to the processing laboratory. To assess HLA-DR cell surface expression by flow cytometry, irradiated SV-BR-1-GM cells cryopreserved in CS5 medium as well as nonirradiated SV-BR-1-GM and A549 cells, both freshly harvested using TrypLE Express, were treated with an Fc receptor blocking agent (Human TruStain FcX™, BioLegend; used 1:20 diluted) and stained with 20 µg/ml of a FITC-conjugated anti-human HLA-DR antibody (clone L243; BioLegend) or with 20 µg/ml of a FITC-conjugated mouse IgG2a, κ isotype control antibody (clone MOPC-173; BioLegend). Stained cells were subjected to flow cytometry, whereby the data was acquired on a BD LSRFortessa™ cell analyzer using FACSDiva software (BD Biosciences, San Jose, CA, USA) and analyzed using Flowing Software version 2.5.1 (Turku Bioimaging, Finland and Turku Centre for Biotechnology, Finland; software developer: Perttu Terho).

### SV-BR-1-GM Peptide Treatment and T Cell Activation

SV-BR-1-GM cells were either serum-starved for 24 h, harvested using trypsin and then irradiated (200 Gy) or cultured in full medium, harvested *via* trypsin and utilized without irradiation. Control human PBMCs (DRB3*01:01 and non-DRB3*01:01) were harvested from fresh whole blood. SV-BR-1-GM cells (serum-starved then irradiated or nonirradiated without serum-starvation) and control PBMCs (irradiated with 50 Gy) were treated with 1 µg/ml (final concentration) of the yellow fewer virus (YFV) Envelope (Env) 43–59 peptide (sequence: ISLETVAIDRPAEVRKV) (35) or of a varicella zoster virus (VZV) open reading frame (ORF) 68 control peptide (Sequence: IWPRNDYDGFLENAHEHHGV) and cocultured with a T cell clone recognizing YFV-DRB3*01:01 peptide-loaded MHCs (pMHCs). No wash-out step of unbound peptides was employed. Prior to coculturing, T cells were rested for 4 days. For coculturing, 50 K SV-BR-1-GM cells or control PBMCs and 50 K rested CD4^+^ T cells per well in 96-well format were used. IFN-γ was assessed from SNs harvested after 72 h of coculturing by ELISA employing anti-human IFN-γ clones B27 and 4S-B3 as capture and detection, respectively, antibodies (both from BioLegend), and DELFIA Europium-labeled streptavidin (PerkinElmer, Waltham, MA, USA) and DELFIA Enhancement Solution (PerkinElmer) for detection using a Perkin Elmer Wallac 1420 Victor2 Microplate Reader.

### HLA Typing and Immunohistochemistry (IHC)

SV-BR-1-GM and peripheral blood cell samples were subjected to high-resolution HLA typing for *HLA-A, HLA-B*, and *HLA-DRB3*. HLA-DRβ3 expression on tumor specimens was assessed on paraffin-embedded tissues by IHC using a rabbit polyclonal antibody raised against an N-terminal region of human HLA-DRβ3 (product code ab196601; Abcam, Cambridge, MA, USA). Both HLA typing and IHC were conducted at the City of Hope (Duarte, CA, USA).

### Frequencies of HLA Allele Combinations

From the allele frequencies (AFs) reported by Gragert et al. (36), estimated “phenotype frequencies” (PFs) were calculated indicating probabilities that an individual carries at least 1 of SV-BR-1-GM’s expressed HLA-A, HLA-B, or HLA-DRB3 alleles (HLA-A*24:02, HLA-B*35:08, HLA-B*55:01, HLA-DRB3*01:01, HLA-DRB3*02:02) or allele groups (HLA-A*24, HLA-B*35, HLA-B*55, HLA-DRB3*01, HLA-DRB3*02). For the following definitions, alleles and allele groups are both referred to as “alleles,” and the sums of the individual SV-BR-1-GM HLA-A, -B, and -DRB3 AFs are referred to as ΣAF_HLA-A_, ΣAF_HLA-B_, and ΣAF_HLA-DRB3_, respectively. The PFs were calculated as follows: PF_HLA-A_ = 1 − (1 − ΣAF_HLA-A_)^2^, PF_HLA-B_ = 1 − (1 − ΣAF_HLA-B_)^2^, and PF_HLA-DRB3_ = 1 − (1 − ΣAF_HLA-DRB3_)^2^, whereby (1-ΣAF_HLA-A_)^2^, (1-ΣAF_HLA-B_)^2^, and (1-ΣAF_HLA-DRB3_)^2^ are the probabilities that an individual does not carry at least 1 of SV-BR-1-GM’s expressed HLA-A, -B, or -DRB3, respectively, alleles (exponent = 2 since diploid, i.e., 2n). AFs used for the calculations were obtained from Gragert et al.’s supplementary data 5 and included frequencies of alleles with different designations but with amino acids identical in the antigen recognition site (Gragert et al.’s supplementary data 1) (36).

### Ethics Approval and Consent to Participate

The clinical aspect of this study was conducted with US FDA and St. Vincent Medical Center institutional review board approval, and written informed patient consent was obtained (16). The clinical trial was registered under ClinicalTrials.gov Identifier NCT00095862.

## Results

### SV-BR-1-GM Samples Used for This Study

**Figure 1:**
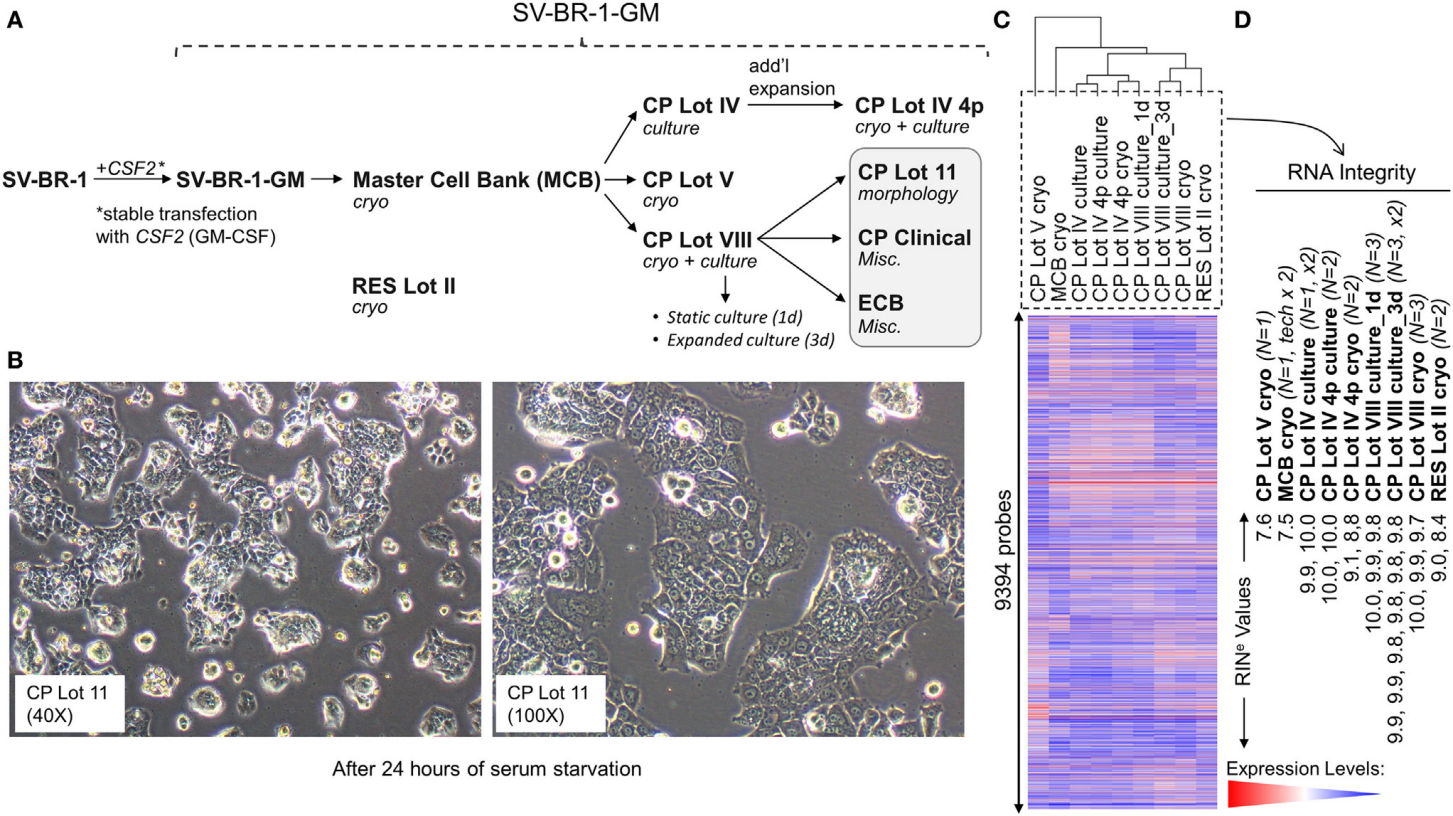
Development of SV-BR-1-GM. **(A)** The SV-BR-1-GM cell line was derived from SV-BR-1 breast cancer cells following stable transfection with *CSF2* [encoding human granulocyte–macrophage colony-stimulating factor (GM-CSF)]. The SV-BR-1 cell line itself was established from a chest wall lesion of a metastatic breast cancer patient (16, 17). The depicted developmental stages of SV-BR-1-GM represent samples used for this study rather than provide a comprehensive overview of all lots generated thus far. From an original master cell bank (MCB), several “clinical product” (CP) lots for actual or potential clinical use were established. “RES Lot II” refers to a research sample type and “ECB” to an engineering cell bank. RNA for gene expression analysis was extracted from cells taken directly from cryogenic vials (“cryo”) or following a culturing step (“culture”). “CP Lot VIII” was studied both as a presumptive static culture (“1d”) and as an expanded culture (“3d”), whereby for the former, samples were harvested on the first day (*t* = 0), and for the latter, on the third day (*t* = 2, i.e., 2 days later) of a time-course assessing GM-CSF secretion (outlined in Figure S5 in Supplementary Presentation S1 in Supplementary Material). Although no culture expansion took place from seeding to *t* = 0 (“static culture”), cell numbers of cultures harvested at *t* = 2 were 1.7–3.2 times the seeding cell numbers (“expanded culture”). **(B)** Culture morphology of SV-BR-1-GM, as exemplified by 40× and 100× original magnifications of a culture derived from the SV-BR-1-GM Lot 11 bank following 24 h of serum starvation. Of note, SV-BR-1-GM cells may grow in monolayers but can also grow as minimally adherent, sphere-like, structures, especially when seeded at very low densities. **(C,D)** Quality control (QC). **(C)** Hierarchical clustering of SV-BR-1-GM samples based on their microarray gene expression profiles. Normalized gene expression levels of samples belonging to the same sample type were averaged (arithmetic mean) prior to clustering. **(D)** Only samples with a RIN^e^ value of at least 7.5 were used for this study. Note that the CP Lot V cryo sample clustered separately and did not pass the minimal variability QC metric (see Materials and Methods) and was thus excluded from additional analyses.

The SV-BR-1 cell line was established from a chest wall lesion of a female metastatic breast cancer patient. The polyclonal SV-BR-1-*GM* cell line was derived from SV-BR-1 cells following stable transfection with *CSF2* (encoding human GM-CSF) and zeocin-selection (US7674456, Patent Application number: US 10/868,094) (16, 17) (Figure 1A). Even though additional parameters are expected to contribute to the potency of SV-BR-1-GM, we assume GM-CSF to be a major factor (15).

Granulocyte–macrophage colony-stimulating factor signaling involves GM-CSF binding to the α subunit of its receptor and recruitment of the receptor’s β subunit (37). Whereas a very restricted region in GM-CSF’s first α helix was suggested to interact with the receptor’s β subunit, several regions further downstream contact the α subunit (38). Compared to the NCBI Reference Sequence of GM-CSF (NP_000749.2), SV-BR-1-GM’s ectopic GM-CSF ORF contains some vector sequence and varies at positions 36 (Thr instead of Met) and 100 (Thr instead of Ile) of the mature GM-CSF protein sequence. However, neither Met36 nor Ile100 seems to be directly involved in receptor binding (38, 39) thus questioning whether these variations actually exert a biological effect, especially impair intracellular signaling. In agreement *with* signaling activity and thus GM-CSF bioactivity, cell culture SN from irradiated SV-BR-1-GM cells supported cell viability and proliferation of MUTZ-3 cells, a cell line reported to depend on cytokines such as GM-CSF (40), whereas SN from parental SV-BR-1 cells (not engineered to express GM-CSF) had at most a minimal effect (data not shown).

Since the excision of the original tumor specimen in 1999, several lots of both SV-BR-1 and SV-BR-1-GM have been manufactured. Figure 1A indicates SV-BR-1-GM samples for which gene expression profiles were generated and their genealogy. Whereas cell banks derived from the MCB (passage 8) and cryopreserved below or at around passage 20 were somewhat arbitrary designated “Clinical Product” (CP) lots, an SV-BR-1-GM sample cryopreserved at passage 30 (RES Lot II) is for this study considered a “research” sample. When used clinically following current practice, SV-BR-1 and SV-BR-1-GM cells are first serum-starved for 24 h in order to remove bovine antigens then irradiated (to abrogate cell proliferation). The serum starvation step was initially carried out prior to cryopreservation of the CP, which was irradiated upon thawing without a preceding culture step. More recently, cell banks have been cryopreserved without prior serum starvation. However, for clinical application, cells from such recent lots are thawed, short-term cultured, serum-starved, then irradiated. As demonstrated in Figure 1B, serum starvation did not obviously perturb epithelial cell morphology as evidenced by the monolayer phenotype with little or no signs of stress. On a related note, nevertheless, it is worth mentioning that at least in some instances, after seeding at (very) low densities in serum-containing medium, SV-BR-1-GM cells were found to grow as minimally adherent, sphere-like, structures. Gene expression profiles were generated on Illumina HumanHT-12 v.4 Bead Chips from RNA either directly obtained from cryopreserved cell suspensions (“cryo” tag in sample names) or from recent cultures (“culture” tag in sample names). Whereas a certain degree of gene expression variability was apparent among different SV-BR-1-GM sample types (Figure 1C), overall, all SV-BR-1-GM samples clustered together and seem to exhibit substantially different gene expression profiles than other established breast cancer cell lines as well as normal breast cell types (Figure S1 in Supplementary Presentation S1 in Supplementary Material). Samples with RNA integrity number equivalent (RIN^e^) values of <7.5 were excluded from the analyses. Similarly, samples, such as CP Lot V cryo, failing another QC test (see Materials and Methods) were not used in further comparative analyses even if their RIN^e^ values may have been ≥7.5 (Figure 1D).

### SV-BR-1-GM Expresses a Gene Signature Associated With Immunostimulatory Functions

We discovered that SV-BR-1-GM cells expressed several genes with known immune system-associated roles, for example, MHC class II-based antigen presentation by professional APCs such as DCs. Among the latter category of genes are *HLA-DMA, HLA-DRA*, and *CD74*, the latter of which giving rise to invariant chain (Ii) and class II-associated invariant chain peptide (CLIP).

**Figure 2:**
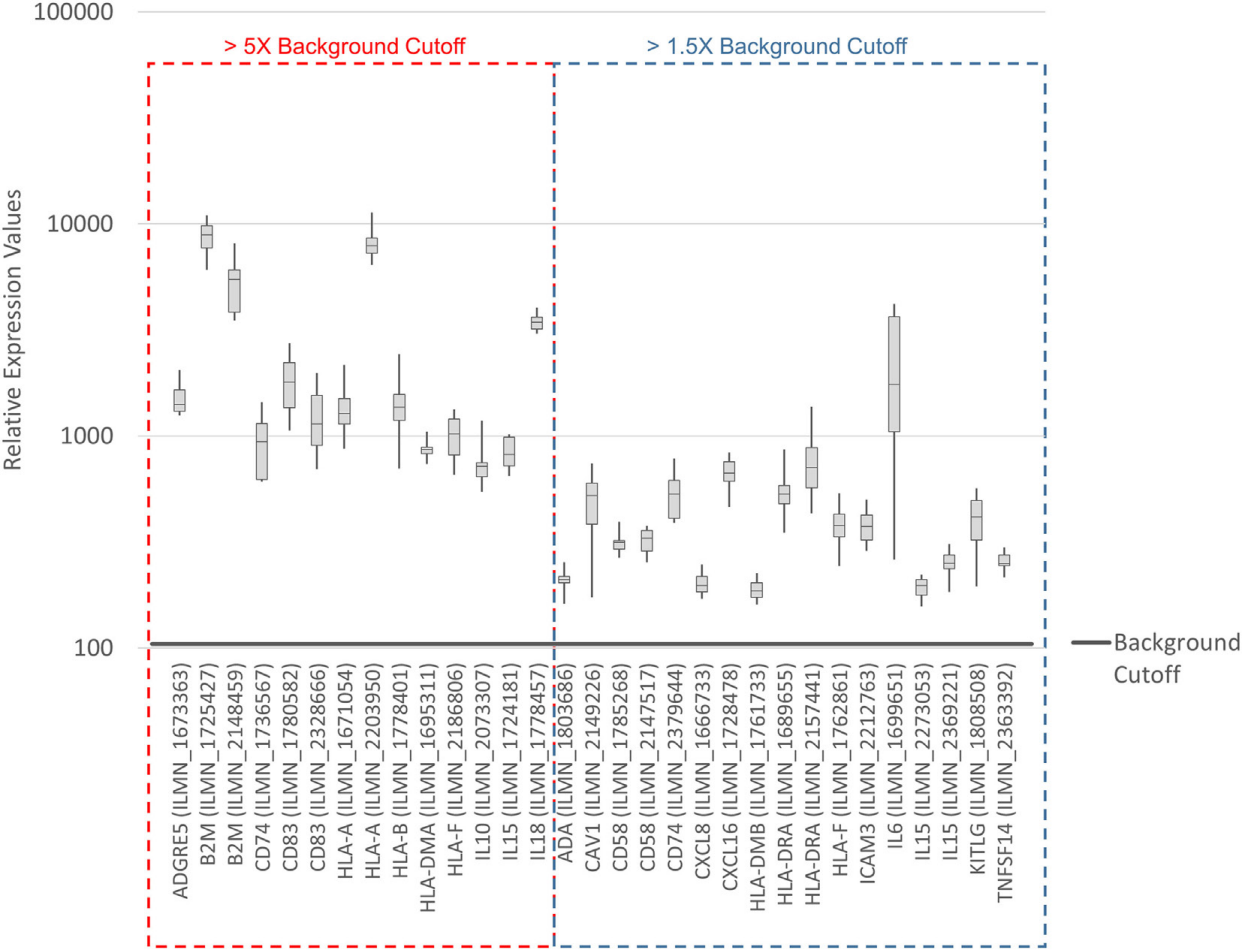
Microarray-based transcript levels of immunostimulatory factors expressed in SV-BR-1-GM cells. 111 genes with known immunostimulatory roles were identified in published reports (41–83) (Data Sheet S3 in Supplementary Material) and their microarray-based RNA expression levels determined. The 22 genes shown presented with transcript levels >1.5 times the background cutoff value (median quantile-normalized “expression” level, see Materials and Methods) in each of the SV-BR-1-GM samples. “Relative Expression Values” refers to quantile-normalized mRNA levels. ILMN_… refer to the Illumina probe sequence identifiers (PROBE_ID) of the probes yielding the expression levels shown.

**Figure 3:**
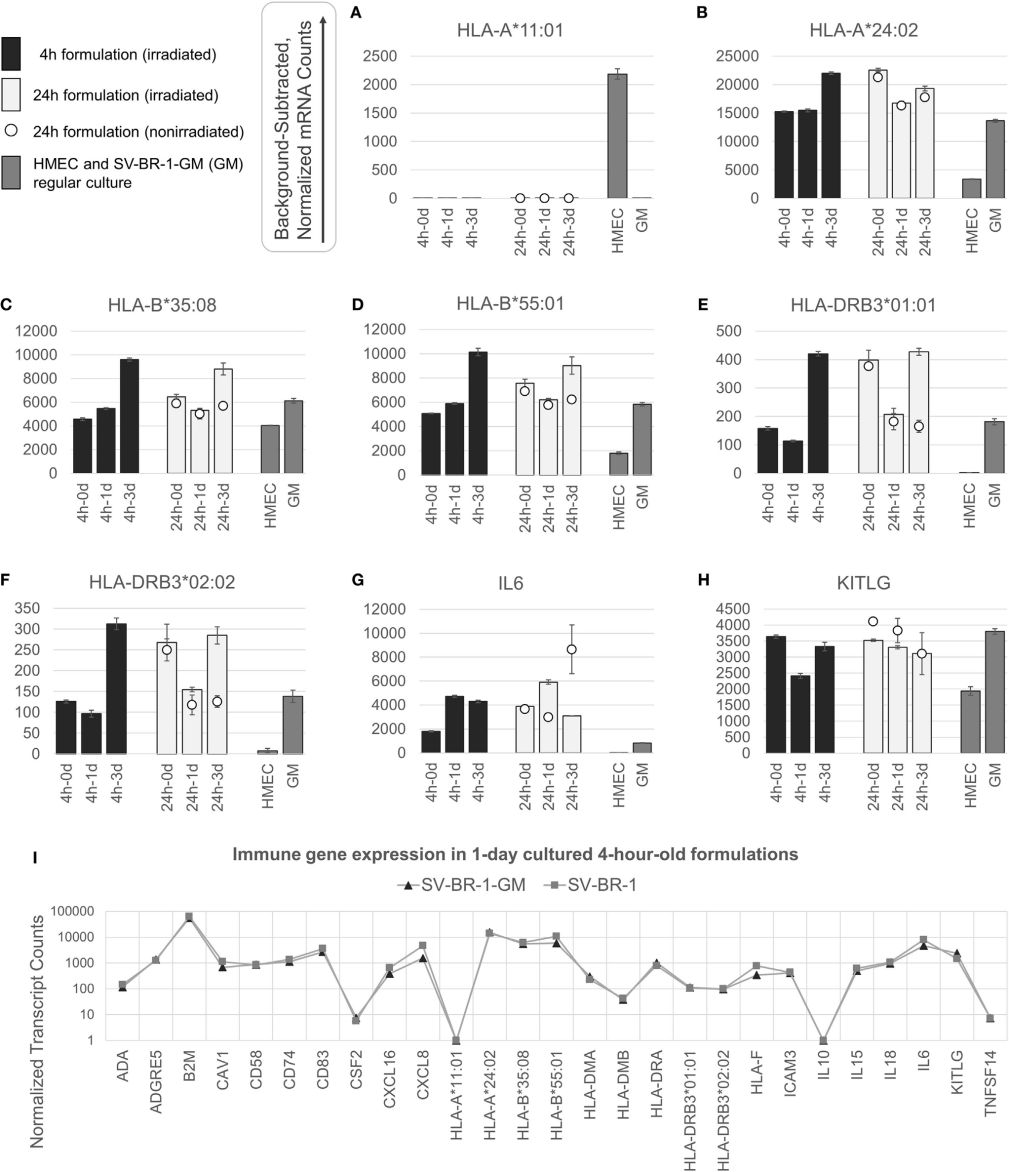
nCounter-based transcript levels of immunostimulatory factors expressed in irradiated and nonirradiated SV-BR-1-GM cells. SV-BR-1-GM cells from 4- and 24-h-old clinical formulations were seeded in 6-well plates and cultured in full medium. Cells were harvested after 1 day and 3 days of culturing then subjected to nCounter-based assessment of the transcript levels of a set of 24 immune-related genes (Immune Signature candidates). For the 24-h series, also nonirradiated cells resuspended in Lactated Ringer’s solution (LRS) and processed in parallel were included. In a separate experiment, transcript levels of the Immune Signature candidates were measured from nonirradiated human mammary epithelial cells (HMECs) and SV-BR-1-GM cells cultured in parallel. Shown are background-subtracted, normalized transcript levels of *HLA-A, -B*, and *-DRB3*, whereby the probes (CodeSets) were designed to distinguish between HLA-A*01:01 **(A)** and HLA-A*24:02 **(B)**, HLA-B*35:08 **(C)** and HLA-A*55:01 **(D)**, and HLA-DRB3*01:01 **(E)** and HLA-DRB3*02:02 **(F)**; and of *IL6* **(G)** and *KITLG* **(H)**. **(I)** Comparison of the expression levels of the 24 immune-related genes between SV-BR-1 and SV-BR-1-GM cells by the nCounter approach. Comparing the 4-h formulations cultured for 1 day, almost identical expression profiles were obtained. Note that the values of *CSF2* (encoding GM-CSF) shown indicate background or minute transcript levels of endogenous *CSF2* since the nCounter ProbeSet employed did not match in sequence with the exogenous *CSF2* expressed by SV-BR-1-GM. For **(A–H)**, values shown are arithmetic means of background-subtracted, normalized transcript levels from triplicate wells ± SDs or from three aliquots per formulation ± SDs for the d0 samples. d0 denotes cells obtained from the original 4- or 24-h-old formulations and lysed without culturing. GM refers to cultured, nonirradiated SV-BR-1-GM cells not subjected to the formulation process. For **(I)**, values shown are arithmetic means of background-subtracted, normalized transcript levels from triplicate (SV-BR-1-GM) or duplicate (SV-BR-1) wells. Values for all genes of the Immune Signature candidates are shown in Data Sheet S3 in Supplementary Material.

To systematically address this observation, we generated a database from published reports (41–83) with 111 genes with known immunostimulatory roles (Data Sheet S3 in Supplementary Material). In particular, genes were included encoding (i) cell surface ligands for T cell costimulatory receptors or other cell surface-associated factors known to positively stimulate T cells (i.e., support T cell activation rather than inhibition), (ii) cytokines and other soluble (free) factors with positive T cell-stimulatory functions such as supporting activation, promoting survival, and/or inducing chemotaxis, (iii) factors promoting maturation, survival, chemotaxis, and/or *in vitro* generation of DCs, and (iv) factors promoting antigen presentation. Of the 111 genes, 22 had quantile-normalized expression levels in all SV-BR-1-GM samples of more than 1.5 times the background cutoff value (see Materials and Methods for definition), with 11 out of these 22 biomarkers expressed at levels more than five times the background cutoff value as demonstrated by at least 1 Illumina probe (Figure 2; Figures S2 and S3 in Supplementary Presentation S1 in Supplementary Material). Of note, microarray-based expression levels of *HLA-DRB3*, even though apparently higher than 1.5 times the background cutoff value, are not shown as we did not consider the corresponding Illumina probe (ILMN_1717261) reliable since it suggested expression in more samples than expected considering *HLA-DRB3* prevalence (data not shown). Nevertheless, *HLA-DRB3 does* seem to be expressed in SV-BR-1-GM cells, as demonstrated by nCounter-based transcript counting (Figure 3).

Among the 22 immune-related biomarkers studied, one salient and unusual finding was the expression of both MHC class I and II components such as *B2M, HLA-A, HLA-B, HLA-F, HLA-E*, and *HLA-H* (MHC class I components) (see Figure S2 in Supplementary Presentation S1 in Supplementary Material), and *HLA-DMA, HLA-DRA*, and *CD74* (MHC class II-associated factors) (see Figures S3 and S4 in Supplementary Presentation S1 in Supplementary Material). Of note, even though *HLA-E* and *HLA-H* were strongly expressed in SV-BR-1-GM cells, they do not seem to be clearly associated with immunostimulatory roles, and therefore are not considered factors likely contributing to the clinical efficacy of SV-BR-1-GM.

**Figure 4:**
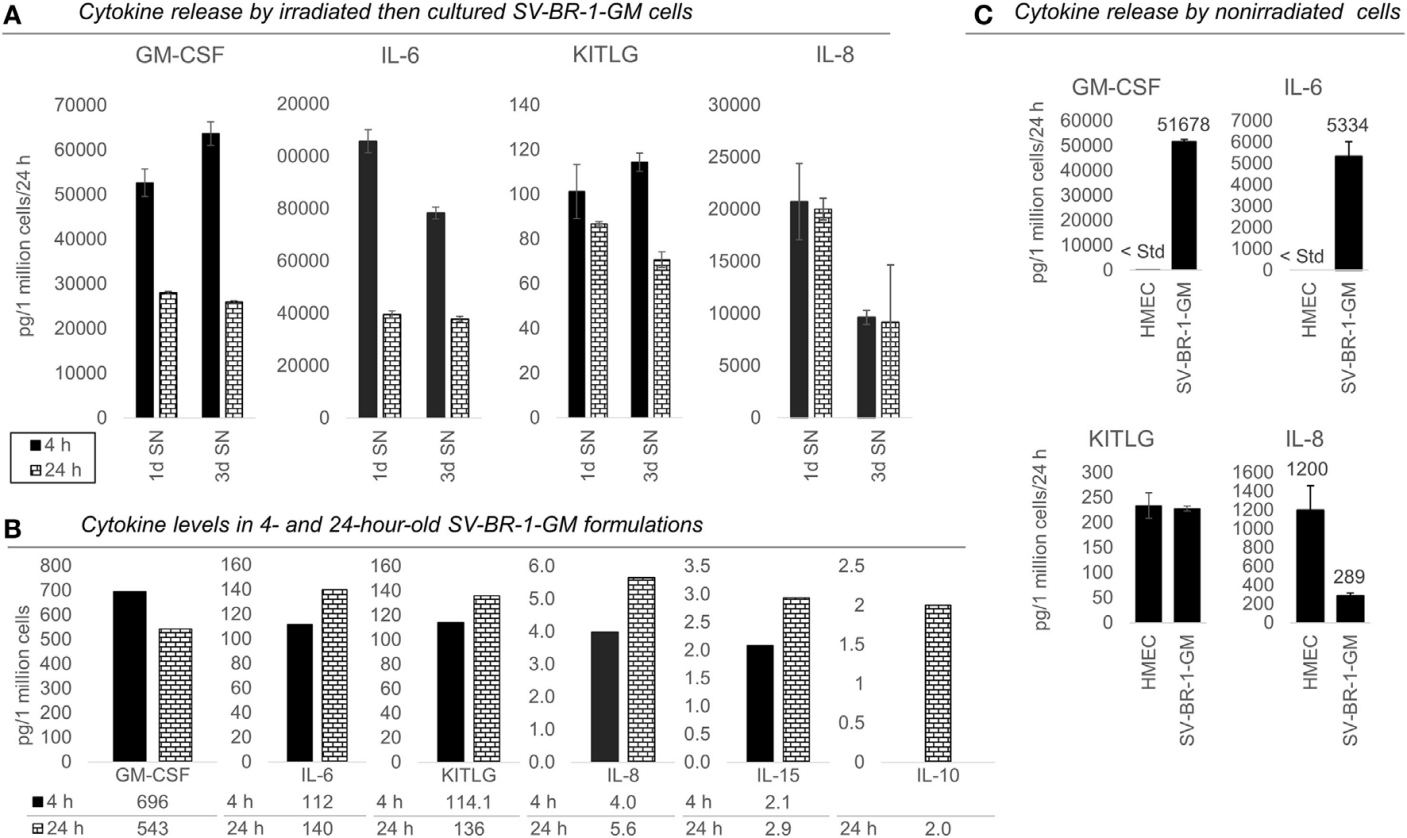
Cytokines secretion by irradiated and nonirradiated SV-BR-1-GM cells. **(A)** SV-BR-1-GM cells from 4- and 24-h-old clinical formulations were seeded in 6-well plates and cultured in full medium. Culture supernatants (SNs) were harvested after 1 day and 3 days of culturing then assessed for cytokine release. Note the substantially reduced levels of granulocyte–macrophage colony-stimulating factor (GM-CSF) and interleukin (IL)-6 for the 24 h compared to the 4 h old formulation. Values shown are arithmetic means from triplicate wells ± SDs, expressed as pg cytokine/1 million viable cells (at time of seeding)/24 h. For KITLG, one of the 24 h, 3d wells was excluded as the obtained cytokine levels were substantially higher than those of the other two wells. For IL-8, ELISAs for the 4 and 24 h samples were conducted on different days. **(B)** Cytokine levels in the Lactated Ringer’s solution (LRS) fractions of the formulations from **(A)**. For IL-10, data are only shown for the 24 h sample. Values shown are arithmetic means from technical duplicates, expressed as pg cytokine/1 million total cells. **(C)** Nonirradiated human mammary epithelial cells (HMECs) and SV-BR-1-GM cells were cultured in parallel then assessed for cytokine release 24 h after replacing the culture medium. Note that the IL-6 and IL-8 levels from the nonirradiated SV-BR-1-GM cells shown here are substantially lower than those from the irradiated SV-BR-1-GM cells shown in **(A)**. Values shown are arithmetic means from triplicate wells ± SDs, expressed as pg cytokine/1 million viable cells/24 h, whereby cell viability was determined on the day of medium change (initiation of cytokine accumulation) from cells cultured in parallel wells.

Intrigued by the possibility that SV-BR-1-GM may have direct immunostimulatory effects beyond those by GM-CSF, we sought to confirm expression of several of the immune-related genes by qRT-PCR. This gene set also included *HLA-DRB3* and *HLA-DMB*, with the latter only barely expressed at more than 1.5 times the background cutoff value but being functionally tied to HLA-DMA (84), another immunostimulatory biomarker expressed in SV-BR-1-GM cells (Figure 2). The confirmatory experiment was conducted on a subset of the SV-BR-1-GM samples used for Illumina microarray analysis and with RNA from MCF7 cells, a breast cancer cell line carrying the HLA-DRB3*02:02 allele (85) as a calibrator sample. As demonstrated in Figure S4 in Supplementary Presentation S1 in Supplementary Material, all MHC class II-related transcripts analyzed (i.e., *HLA-DRA, HLA-DRB3, HLA-DMA, HLA-DMB, CD74*) were not only expressed in SV-BR-1-GM cells *per se* but this at substantially higher levels than in MCF7 cells. Furthermore, even though SV-BR-1-GM cells were engineered to express *CSF2* (encoding GM-CSF), we did not detect *CSF2* transcripts by Illumina microarray gene expression profiling (data not shown). However, this finding is not surprising because the Illumina probe for *CSF2* (ILMN_1661861) mapped to a sequence in the gene’s 3′ untranslated region which is not represented by SV-BR-1-GM’s ectopic *CSF2* sequence. Importantly, by ELISA, we did demonstrate GM-CSF protein expression in medium conditioned by both irradiated and nonirradiated SV-BR-1-GM cells (Figure 4; Figure S5 in Supplementary Presentation S1 in Supplementary Material).

Taken together, in addition to the 22 genes with transcript representation in Figure 2, also *HLA-DRB3* and *CSF2* (GM-CSF) are considered relevant immunostimulatory factors potentially contributing to the efficacy of the SV-BR-1-GM targeted immunotherapy, raising the roster of queried immune-related factors to 24.

### Verification of Expression of the 24 Immune-Related Factors

To establish whether the 24 genes with immunostimulatory functions are also expressed in SV-BR-1-GM cells having undergone the clinical formulation process (which includes irradiation of the cells then resuspension in LRS), cells were formulated at the UC Davis GMP facility (Sacramento, CA, USA) then courier-transported under temperature controlled (2–8°C) conditions to the processing laboratory in Berkeley, CA, USA. The shipping containers were opened then samples processed, 4 h (4) and 24 h (24) after completion of the formulation process. This mirrored the actual protocol activities closely: The 4-h time point represents the approximate time from completion of the formulation process to inoculation for patients dosed at a clinical site in vicinity of the UC Davis GMP facility. The 24-h time point represents the formulations’ expiry time.

Since in a clinical context, SV-BR-1-GM cells are inoculated as a replication-incompetent (irradiated) cell suspension with variable percentages of live cells, it is reasonable to speculate that continued expression of the immune-related genes improves SV-BR-1-GM’s therapeutic efficacy. To assess whether these genes are indeed still expressed in cells having undergone the formulation process, cells and SNs from the 4- and 24-h-old formulations were either analyzed directly (without culturing) or after a cell culturing period of 1 or 3 days. Whereas the original LRS and culture SNs were analyzed for presence and levels of secreted immune-related factors by ELISA, we used nCounter-based transcript counting to assess transcript levels of all 24 immune-related genes.

Appreciable levels of RNA from all but three (*IL10, TNFSF14*, and endogenous *CSF2*) of the immune-related genes were observed in samples derived from the clinical formulations, both from formulated cells cultured prior to harvest and from cell aliquots of the formulations lysed without culturing (Figure 3 and Data Sheet S3 in Supplementary Material), consistent with continued transcription of most of these immune-related genes despite the irradiation step. The lack of more than at most minimal endogenous *CSF2* (encoding GM-CSF) transcript levels is not concerning, since the nCounter CodeSet employed was not designed to detect transcripts from the ectopic (transfected) *CSF2* ORF. However, in the case of HLA-A, only one of SV-BR-1-GM’s alleles (HLA-A*24:02) seems to be expressed. Since the nCounter CodeSet (probe) for the other, HLA-A*11:01, did yield a clear signal with another cell type, HMECs, we have no evidence suggesting lack of probe hybridization *per se* (Figures 3A,B), although we have not experimentally confirmed the hybridization efficiency of the CodeSet. Expression of *HLA-B* and *HLA-DRB3* was apparent for each of SV-BR-1-GM’s alleles: HLA-B*35:08, HLA-B*55:01, HLA-DRB3*01:01, and HLA-DRB3*02:02 (Figures 3C–F). Furthermore, substantial RNA expression levels were observed for *IL6* and *KITLG* (Figures 3G,H). To compare expression levels of the 24 immune-related genes between parental SV-BR-1 cells and SV-BR-1-GM cells, also SV-BR-1 cells were analyzed by the nCounter approach. Almost identical expression profiles were obtained with 4-h-old formulations cultured for 1 day (Figure 3I). This is consistent with the assumption that the engineering of the SV-BR-1 cells to express GM-CSF did not affect the cells’ ability to exert immunostimulatory functions. Furthermore, based on our microarray data, SV-BR-1-GM cells at most marginally express *CSF2RB*, encoding the β chain common for the GM-CSF, IL-3, and IL-5 receptors, suggesting lack of autocrine GM-CSF effects (data not shown) (37).

Also, we confirmed *protein* expression of several of the immune-related factors. GM-CSF, IL-6, IL-8, and KITLG (free SCF) were detected in SNs from cultured formulations (Figure 4A) and in the original 4- and 24-h formulations (Figure 4B). However, IL-10 and IL-15 were not detected in culture SNs (data not shown), but low levels were measured in the original LRS-based formulations (Figure 4B). Nevertheless, these data suggest only minimal, if any, contribution of IL-10 and IL-15 to the clinical activity of SV-BR-1-GM. In contrast to *IL15*, transcript levels of *IL10* could not be verified (Data Sheet S3 in Supplementary Material), suggesting that indeed at most minute levels of IL-10 are expressed by SV-BR-1-GM cells. Therefore, IL-10 is not expected to contribute to SV-BR-1-GM’s MoA. Moreover, while IL-18 was detected by two different ELISA kits from two different vendors, the presumptive protein levels were highly discordant. Whereas high IL-18 levels were measured by one kit in (i) the original formulations (LRS), (ii) SN from cultured and irradiated SV-BR-1-GM cells, and (iii) SN from cultured and nonirradiated SV-BR-1-GM cells, IL-18 was only detected in LRS at the highest sample concentration tested (1:10 diluted) by the other kit (data not shown).

To address which of the immune-related factors are overexpressed in SV-BR-1-GM cells compared to normal breast cells, RNA and protein levels were measured from both nonirradiated SV-BR-1-GM and nonirradiated HMECs cultured in parallel. Similarly to the formulations, for SV-BR-1-GM cells, clear transcript expression was confirmed for all 24 genes except for *IL10* and *TNFSF14*. Importantly, even though several genes were expressed at higher levels in HMECs than in SV-BR-1-GM cells, transcript levels of all MHC class II-associated genes (*HLA-DMA, -DMB, -DRA, -DRB3*, and *CD74*) were considerably higher in SV-BR-1-GM cells compared to HMECs (Figure 3; Data Sheet S3 in Supplementary Material). Furthermore, whereas GM-CSF, IL-6, IL-8, and KITLG were measured and detected in SV-BR-1-GM culture SNs, GM-CSF and IL-6 were not (or at most at minute levels) detected in HMEC SNs. On the other hand, free SCF (KITLG) levels were similar in SV-BR-1-GM and HMEC SNs, but IL-8 levels were >4 times higher in SNs from HMECs than from SV-BR-1-GM cells (Figure 4C).

**Figure 5:**
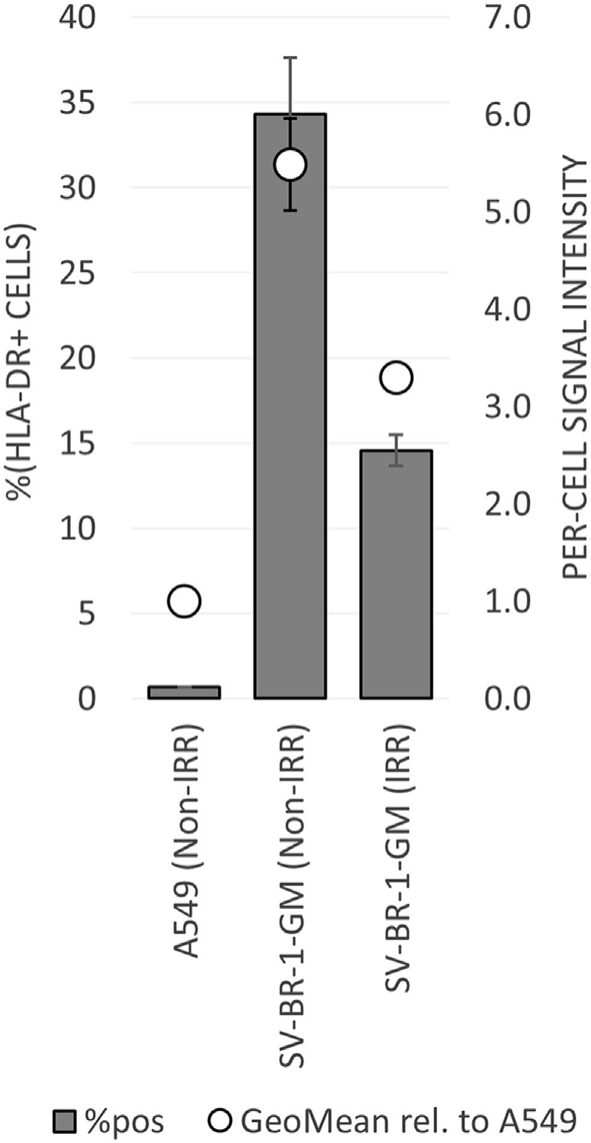
HLA-DR expression on irradiated and nonirradiated SV-BR-1-GM cells. SV-BR-1-GM cells were stained with a FITC-conjugated mouse monoclonal anti-HLA-DR antibody (clone L243) recognizing an epitope from HLA-DRα only present when complexed with an HLA-DRβ chain. Shown are arithmetic means ± SDs of technical triplicates (SV-BR-1-GM) or duplicates (A549) from (i) the percentages of HLA-DR positive cells (left axis) and (ii) the per-cell HLA-DR signal intensities normalized to the geometric mean of the signal intensity by the A549 negative control and reference cell line (value of A549 = 1) (right axis) (86, 87) Non-IRR, non-irradiated; IRR, irradiated.

To establish whether and to what extent HLA-DRβ3 (encoded by *HLA-DRB3*) is expressed on the surface of SV-BR-1-GM cells, cells were irradiated then cryopreserved in CS5 freeze medium. Both cryopreserved and freshly harvested, nonirradiated SV-BR-1-GM cells were subjected to flow cytometry employing a monoclonal anti-HLA-DR antibody (clone L243) recognizing a conformational epitope on HLA-DRα only present when complexed with an HLA-DRβ chain. In the case of SV-BR-1-GM, the β chain is thought to be HLA-DRβ3 for the following reasons: (i), *HLA-DRB1* is not (or at most marginally) expressed at the transcript level (Data Sheet S3 in Supplementary Material); (ii), SV-BR-1-GM’s diploid presence of *HLA-DRB3* indicates absence of *HLA-DRB4* and *5*; and (iii), the remaining *HLA-DRB* genes (*HLA-DRB2, 6, 7, 8*, and *9*) are pseudogenes (73, 85). As demonstrated in Figure 5, HLA-DR expression was heterogeneous, with about a third of the nonirradiated and some 15% of the irradiated SV-BR-1-GM cells staining positive for HLA-DR. Similarly, the per-cell signal intensity was higher in nonirradiated than in irradiated cells, suggesting that the irradiation process negatively affected HLA-DR gene expression and/or transport to the cell surface. A549 cells served as negative control and reference cell line (86, 87).

**Figure 6:**
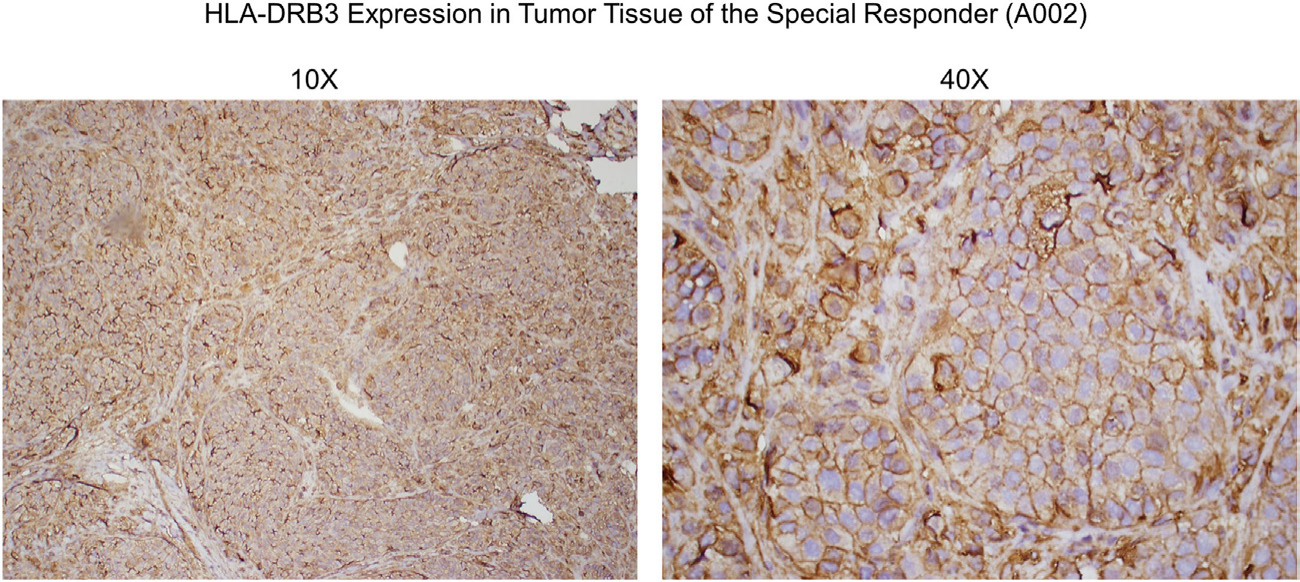
Anti-HLA-DRβ3 antibody staining of a tumor specimen from the strong clinical responder. To assess whether the strong clinical responder (16), referred to as subject A002 in this article, presented with tumor expression of HLA-DRβ3, paraffin-embedded A002 tumor material was stained with a rabbit polyclonal antibody raised against an N-terminal region of human HLA-DRβ3. As demonstrated, immunoreactivity was apparent.

In collaboration with Creatv MicroTech (Potomac, MD, USA), in a preliminary study, nonirradiated SV-BR-1-GM cells were fixed and stained with a polyclonal anti-HLA-DRβ3 antibody (product code ab196601; Abcam) then analyzed in the context of an IHC-like process. Of note, given the high sequence similarity among HLA-DRβ chains, it seems likely that this antibody (which was also used to generate the data shown in Figure 6) can crossreact with HLA-DRβ chains other than β3. 15% of the cells were strongly positive for HLA-DRβ3, and another 21% had an intermediate signal (preliminary data not shown). However, while 0% of the negative control human umbilical vein endothelial cells (HUVECs) cells were strongly HLA-DRβ3 positive, 19% of the HUVECs had an intermediate signal (preliminary data not shown). Nevertheless, these findings also suggest that at least about 15 percent of SV-BR-1-GM cells express HLA-DR and that the β chain in these HLA-DR complexes is β3, since, based on our RNA and HLA typing data, SV-BR-1-GM cells do not express appreciable levels of another HLA-DRβ chain.

In preliminary flow cytometry experiments with nonirradiated SV-BR-1-GM cells, we also attempted to measure the cell surface levels of HLA-DRβ3 directly using a mouse polyclonal antibody raised against the full-length protein (MaxPab B02P; Abnova, Taipei, Taiwan). With this antibody, a very small (~3%) fraction of apparently HLA-DRβ3 (very weakly) positive SV-BR-1-GM cells was identified, with the negative control cell lines (A549 and T-47D) being negative (data not shown). However, given that the HLA-DRβ3 per-cell signal intensity was rather minuscule, the validity of this result is questioned, especially in light of the data both obtained with the anti-HLA-DRα antibody (Figure 5) and by Creatv MicroTech.

Taken together, we have confirmed expression of 22 out of the 24 immune-related genes considered relevant for SV-BR-1-GM’s MoA. The complete 22-gene Immune Signature is shown in Table 1.

**Table 1:** 22-gene Immune Signature expressed in SV-BR-1-GM cells.

Gene symbol	Official full name/description	Aliases
ADA	Adenosine deaminase	
ADGRE5	Adhesion G protein-coupled receptor E5	CD97, TM7LN1
B2M	Beta-2-microglobulin	IMD43
CAV1	Caveolin 1	BSCL3, CGL3, LCCNS, MSTP085, PPH3, VIP21
CD58	CD58 molecule	LFA-3, LFA3, ag3
CD74	CD74 molecule; invariant chain and CLIP	DHLAG, HLADG, II, Ia-GAMMA
CD83	CD83 molecule	BL11, HB15
CSF2	Colony-stimulating factor 2	GMCSF
CXCL8	C-X-C motif chemokine ligand 8	GCP-1, GCP1, IL8, LECT, LUCT, LYNAP, MDNCF, MONAP, NAF, NAP-1, NAP1
CXCL16	C-X-C motif chemokine ligand 16	CXCLG16, SR-PSOX, SRPSOX
HLA-A	Major histocompatibility complex, class I, A	HLAA
HLA-B	Major histocompatibility complex, class I, B	AS, B-4901, HLAB
HLA-DMA	Major histocompatibility complex, class II, DM alpha	D6S222E, DMA, HLADM, RING6
HLA-DMB	Major histocompatibility complex, class II, DM beta	D6S221E, RING7
HLA-DRA	Major histocompatibility complex, class II, DR alpha	HLA-DRA1, MLRW
HLA-DRB3	Major histocompatibility complex, class II, DR beta 3	HLA-DR1B, HLA-DR3B
HLA-F	Major histocompatibility complex, class I, F	CDA12, HLA-5.4, HLA-CDA12, HLAF
ICAM3	Intercellular adhesion molecule 3	CD50, CDW50, ICAM-R
IL6	Interleukin 6	BSF-2, BSF2, CDF, HGF, HSF, IFN-beta-2, IFNB2, IL-6
IL15	Interleukin 15	IL-15
IL18	Interleukin 18	IGIF, IL-18, IL-1g, IL1F4
KITLG	KIT ligand	DCUA, DFNA69, FPH2, FPHH, KL-1, Kitl, MGF, SCF, SF, SHEP7

*Genes with immunostimulatory roles expressed in SV-BR-1-GM cells. Gene symbols refer to the NCBI designations and HUGO Gene Nomenclature Committee (HGNC) recommendations. Gene symbols, official full names/descriptions, and aliases are indicated as shown on the respective NCBI Gene sites with or without additional information*.

### HLA Allele Matches Between SV-BR-1-GM and a Robust Clinical Responder

By low-resolution HLA typing, we previously established that the robust clinical responder (here referred to as subject A002) and SV-BR-1-GM cells had similarities in their HLA phenotypes (16). To find out whether such similarities are further reflected at the allele level, peripheral blood cells from patients and SV-BR-1-GM cells were subjected to high-resolution HLA typing for *HLA-A, -B*, and *-DRB3*. Indeed, whereas the three clinical trial subjects who did not experience SV-BR-1-GM-induced tumor regression had at most an HLA-A allele match with SV-BR-1-GM, subject A002 matched both at HLA-A (*11:01) and HLA-DRB3 (*02:02) (Table 2). However, since we cannot confirm expression of HLA-A*11:01 in SV-BR-1-GM cells with the nCounter CodeSet (probe) employed (Figure 3A), in this patient, the HLA-DRB3*02:02 match alone may have been clinically relevant. This finding agrees with an MoA in which tumor antigens displayed on HLA-DRβ3-based MHCs expressed on SV-BR-1-GM cells would contribute to the therapeutic efficacy of the cell line.

**Table 2:** HLA alleles.

Subject ID	Cancer Dx	Survival (months)	Tumor regression	HLA-A		HLA-B		HLA-DRB3	
A001	Breast	40.7	No	02:01	**24:02^+^**	13:02	41:01	03:01	–
A002	Breast	33.7	Yes	02:01	11:01**^+^**	18:03	44:02	**02:02^+^**	–
A003	Ovarian	35.6	No	02:01	03:01	07:02	13:02	Neg.	–
B001	Breast	7.0	No	11:01^+^	–	35:01^(+)^	40:01	Neg.	–
SV-BR-1-GM	N/A	N/A	N/A	11:01	**24:02**	**35:08**	**55:01**	**01:01**	**02:02**

*HLA alleles of SV-BR-1-GM and peripheral blood cells from four study subjects (ClinicalTrials.gov Identifier: NCT00095862) were identified. Subject A002, with both *HLA-A* and *HLA-DRB3* allele matches to SV-BR-1-GM, responded to SV-BR-1-GM inoculation with substantial tumor regression [16]. “+” indicates allele level and “(+)” allele *group* level identity with SV-BR-1-GM. Alleles expressed in SV-BR-1-GM cells are listed in bold*.

Since HLA typing was conducted using peripheral blood cells, and because targeted cancer immunotherapy requires cancer cell MHC expression, we sought to identify whether HLA-DRβ3 protein is present on a paraffin-embedded tumor specimen from clinical trial subject A002. As demonstrated in Figure 6, immunoreactivity was indeed apparent, thus further supporting the postulated role of MHC class II in the MoA of SV-BR-1-GM.

### CTAs Expressed in SV-BR-1-GM

Cancer/testis antigens represent a class of antigens with physiological expression predominantly restricted to testicular or placental tissue, and, for a subset, brain tissue. However, CTAs may become upregulated following malignant conversion of cells from a variety of organs. For many of such CTAs, immunostimulatory roles have been established (6, 32–34, 88–91).

**Figure 7:**
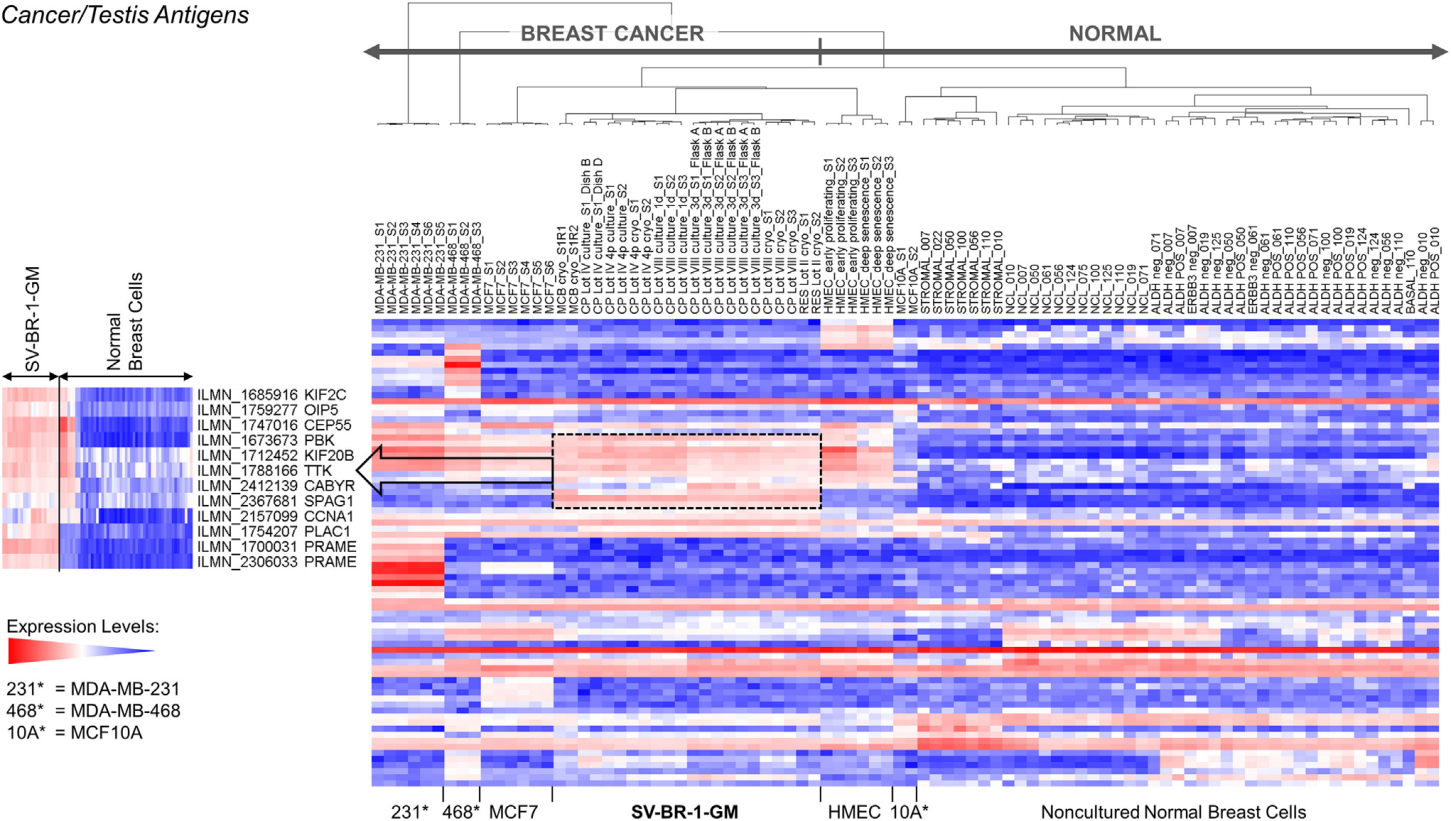
Cancer/testis antigen (CTA) expression in SV-BR-1-GM cells. RNA expression levels of 279 confirmed or putative CTAs (Data Sheets S4 in Supplementary Material) were compared between SV-BR-1-GM, other established breast cancer cell lines, and several normal human breast cell types. Quantile-normalized and log2-transformed microarray-based RNA expression levels are displayed according to a global gradient color scheme. Red means a higher expression level than white, and white means a higher expression level than blue. Only CTAs with a maximum representative expression value among all samples >1.5 times the background cutoff value were included.

**Figure 8:**
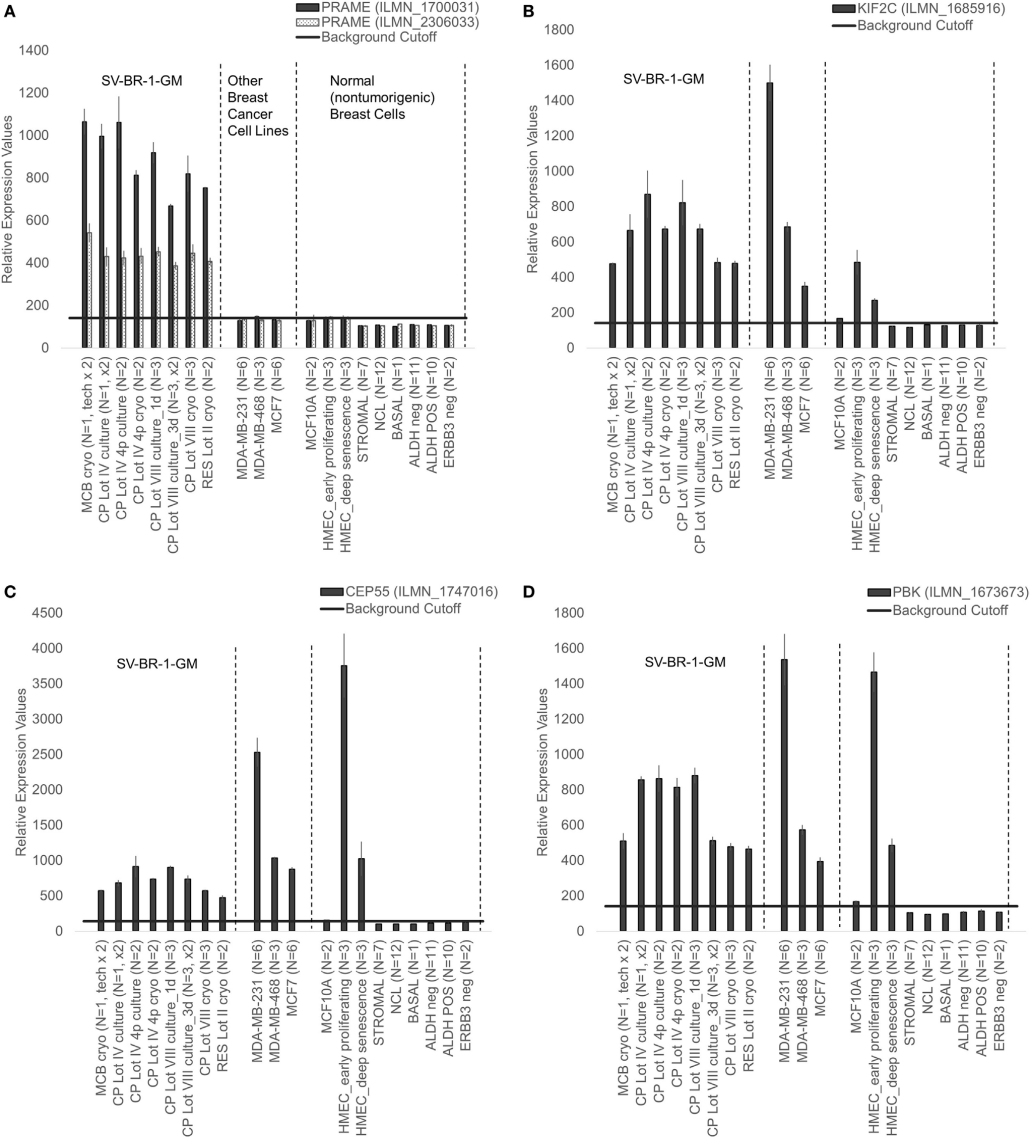
Cancer/testis antigen selectively expressed in SV-BR-1-GM cells. Quantile-normalized microarray-based RNA levels of *PRAME* **(A)**, *KIF2C* **(B)**, *CEP55* **(C)**, and *PBK* **(D)** “Relative Expression Values” refers to quantile-normalized mRNA levels. Values shown are arithmetic means. Error bars indicate SDs, except for STROMAL, NCL, BASAL, ALDH neg, ALDH POS, ERBB3 neg. for which standard error of the mean (S.E.M.) values are shown.

Given such features, we assessed the mRNA expression levels of 279 confirmed or putative CTAs (Data Sheets S4 in Supplemental Material) in SV-BR-1-GM cells in comparison to several other breast cancer cell lines and normal breast cells. Following hierarchical clustering on both genes and samples, a group of CTA genes (*KIF2C, OIP5, CEP55, PBK, KIF20B, TTK, CABYR, SPAG1, CCNA1, PLAC1*, and *PRAME*) emerged with particularly good discrimination between SV-BR-1-GM and normal breast cells (Figure 7). However, expression of some of the genes in SV-BR-1-GM cells was weak and/or highly variable among the different SV-BR-1-GM samples. Nevertheless, *PRAME, KIF2C, CEP55*, and *PBK* were robustly expressed in SV-BR-1-GM cells (Figure 8) with *PRAME* exhibiting the highest fold-change ratio between the SV-BR-1-GM expression level and the maximum expression level among the normal breast samples (Table 3). In contrast to *PRAME* (Figure 8A), *KIF2C, CEP55*, and *PBK* were also expressed in cultured HMECs. Interestingly, the expression values of the latter three genes are higher in “early proliferating” than in senescent HMECs (Figures 8B–D) (30), suggesting that perhaps also *in vivo* proliferating breast epithelial cells express these genes. A list of CTAs with transcript expression values greater in SV-BR-1-GM cells than in normal breast cell types is shown in Table 3.

**Table 3:** Cancer/testis antigen expression in SV-BR-1-GM cells.

Gene symbol (NCBI)	Probe identifier	SV-BR-1-GM	Max. expression values (among nonmalignant cells)		SV-BR-1-GM/Max	
	
			C + NC	NC	C + NC	NC
PRAME	ILMN_1700031	869.1	142.8	114.7	6.1	7.6
PRAME	ILMN_2306033	431.9	145.1	112.5	3.0	3.8
PBK	ILMN_1673673	663.0	1465.5	107.2	0.5	6.2
CEP55	ILMN_1747016	708.4	3756.0	126.2	0.2	5.6
KIF2C	ILMN_1685916	668.0	484.9	130.8	1.4	5.1
PLAC1	ILMN_1754207	415.4	150.1	144.9	2.8	2.9
OIP5	ILMN_1759277	405.9	372.3	167.3	1.1	2.4
CABYR	ILMN_2412139	369.6	533.8	179.0	0.7	2.1
SPAG1	ILMN_1712773	289.1	203.7	181.6	1.4	1.6

*Max refers to the maximum transcript level among the representative values of each non-cultured (NC) sample type. SV-BR-1-GM/Max refers to the quotients between the representative SV-BR-1-GM expression values and the Max values (see Materials and Methods for details). Note that for *PBK, CEP55*, and *CABYR*, SV-BR-1-GM/Max is >1 with the non-cultured (NC) cell types alone, but is <1 when including the cultured (C) breast cells (C + NC). This may suggest that culturing can upregulate expression of these genes. C: cultured normal cell types: MCF10A from GEO Data Set GSE48398, and early_proliferating and deep_senescence human mammary epithelial cells (HMEC) from GSE56718 (30); NC: non-cultured normal cell types: ALDH NEG, ALDH POS, ERBB3 NEG, NCL, BASAL, STROMAL from GSE35399 (31). Background cutoff value: 141.16*.

### Other Candidate Immunogens Expressed in SV-BR-1-GM Cells

Even though SV-BR-1-GM expresses an “immune signature” (Table 1), the latter alone is unlikely sufficient to induce a strong tumor-directed immune response as it does not provide cancer specificity. It is reasonable to hypothesize that for patients responding to whole-cell targeted cancer immunotherapies with tumor regression such missing directionality is provided by the cell line through overexpression of TAAs coexpressed in the tumors. Candidate TAAs for the SV-BR-1-GM cell line include the CTAs addressed above and illustrated in Figures 7 and 8.

To systematically search for SV-BR-1-GM antigens with potential to break immune tolerance by overexpression, we employed a 2-tier microarray-based approach. First, genes upregulated in SV-BR-1-GM cells relative to normal breast cells were identified. For this, we compared gene expression levels in SV-BR-1-GM cells to those of a variety of normal human breast cell types described by Shehata et al. [GEO DataSet GSE35399 (31)], Lowe et al. [GEO DataSet GSE56718 (30)], and MCF10A from GEO DataSet GSE48398. Two serial filters were applied to quantile-normalized gene expression values to enrich for genes likely differentiating SV-BR-1-GM from normal breast cells. After low-stringency filtration, 588 different genes (including some of non-coding RNA) were retained, of which, after medium-stringency filtration, 353 remained (Figures S6 and S7 in Supplementary Presentation S1 in Supplementary Material; Data Sheet S5 in Supplementary Material).

Second, among the 353 genes retained after medium-stringency filtration, those not only upregulated relative to normal breast cells but also relative to tissues other than breast were considered *verified* immunogen candidates. This second criterion is sought to enrich for genes with an “actual” potential to break immune tolerance since physiologically high levels of gene expression not only in breast but also in tissues of other organs may prevent breakage of tolerance. The high-stringency filter applied in this step compared GEO DataSet GSE29431 (breast cancer tissues) to a subset of samples represented by GEO DataSet GSE7307 (nonmalignant tissues) (Data Sheet S1 in Supplementary Material) and was conducted on 328 genes retained after medium-stringency filtration (no Affymetrix probes were found for 25 of the 353 genes retained after medium-stringency filtration). Of note, the filter cutoff criteria (see Materials and Methods) were selected to retain *ERBB2* (*HER2*/*neu*), whose immunogenic properties are being explored in several clinical trials (13, 92). Thirty-one genes were verified as TAA candidates using this strategy (Table 4). Interestingly, four of them, *ERBB2* (*HER2*/*neu*), *MIEN1, PGAP3*, and *STARD3*, mapped to 17q12, a region frequently amplified in breast cancer (Figure S8 in Supplementary Presentation S1 in Supplementary Material). In the context of breast cancer, ERBB2 (HER2) is clearly the most widely studied TAA among these four candidate immunogens. However, the apparent coregulation of the other three with *ERBB2* suggests that Her2 positive tumors (likely also expressing one or several of the other antigens) may be effectively targeted by SV-BR-1-GM targeted immunotherapy by means of antigens beyond ERBB2.

**Table 4:** *In silico* verified candidate TAAs.

Gene symbol	Description/official full name	Location	Affymetrix probe ID	Score
ALG8	Alpha-1,3-glucosyltransferase	11q14.1	203545_at	3.69
ARPC5L	Actin-related protein 2/3 complex, subunit 5 like	9q33.3	226914_at	3.96
AZIN1	Antizyme inhibitor 1	8q22.3	212461_at	3.28
CBX2	Chromobox 2	17q25.3	226473_at	5.65
CENPN	Centromere protein N	16q23.2	222118_at	3.12
COL8A1	Collagen type VIII alpha 1 chain	3q12.1	226237_at	10.44
DCAF10	DDB1 and CUL4-associated factor 10	9p13.2	230679_at	8.75
			226511_at	3.84
EIF3H	Eukaryotic translation initiation factor 3 subunit H	8q23.3-q24.11	230570_at	6.87
ERBB2	erb-b2 receptor tyrosine kinase 2	**17q12**	234354_x_at	11.70
			216836_s_at	3.95
HIST1H4H	Histone cluster 1 H4 family member h	6p22.2	232035_at	11.26
			208180_s_at	7.57
IGFBP5	Insulin-like growth factor binding protein 5	2q35	1555997_s_at	3.52
INTS7	Integrator complex subunit 7	1q32.3	218783_at	4.30
KRT19	Keratin 19	17q21.2	228491_at	9.24
KRT81	Keratin 81	12q13.13	213711_at	6.76
MGAT4A	Mannosyl (alpha-1,3-)-glycoprotein beta-1,4-N-acetylglucosaminyltransferase, isozyme A	2q11.2	231283_at	5.85
			226039_at	3.62
			219797_at	3.51
MIEN1	Migration and invasion enhancer 1	**17q12**	224447_s_at	6.40
MTHFD2	Methylenetetrahydrofolate dehydrogenase (NADP+ dependent) 2, methenyltetrahydrofolate cyclohydrolase	2p13.1	201761_at	3.01
PAK1	p21 (RAC1)-activated kinase 1	11q13.5-q14.1	230100_x_at	3.18
PDCD6	Programmed cell death 6	5p15.33	222380_s_at	3.13
PDRG1	p53 and DNA damage regulated 1	20q11.21	225075_at	3.52
PGAP3	Post-GPI attachment to proteins 3	**17q12**	221811_at	5.92
			55616_at	5.14
PIGK	Phosphatidylinositol glycan anchor biosynthesis class K	1p31.1	209707_at	3.03
RFC5	Replication factor C subunit 5	12q24.23	203209_at	3.58
RSF1	Remodeling and spacing factor 1	11q14.1	222541_at	8.91
			229885_at	4.33
SHB	SH2 domain containing adaptor protein B	9p13.1	1557458_s_at	4.11
SLC35A2	Solute carrier family 35 member A2	Xp11.23	209326_at	5.47
STARD3	StAR-related lipid transfer domain containing 3	**17q12**	202991_at	3.50
SYNE4	Spectrin repeat containing nuclear envelope family member 4	19q13.12	235515_at	4.00
TNPO1	Transportin 1	5q13.2	1557278_s_at	3.71
			225765_at	3.34
UBR5	Ubiquitin protein ligase E3 component n-recognin 5	8q22.3	208882_s_at	3.09
XPOT	Exportin for tRNA	12q14.2	212160_at	3.00

*Genes retained after high-stringency filtration, i.e., genes “verified” on breast cancer and nonmalignant tissue data sets. “Score” refers to the quotient of the 95th percentile of the quantile-normalized breast cancer samples in GSE2943 and the 95th percentile of the quantile-normalized and grouped normal tissues in GEO DataSet GSE7307, whereby the maximum expression value across all samples per group was assigned to the group. An arbitrary score of 3.00 was selected as cutoff for the genes listed. The sample IDs of the different groups of normal tissues are shown in Data Sheet S1 in Supplementary Material. Gene symbols refer to the NCBI designations and HUGO Gene Nomenclature Committee (HGNC) recommendations. Official gene symbols, descriptions/official full names, and locations are indicated as shown on the respective NCBI Gene sites. 17q12, the location of ERBB2, MIEN1, PGAP3, and STARD3, is marked in bold face to emphasize proximity*.

### SV-BR-1-GM Cells Are APCs

**Figure 9:**
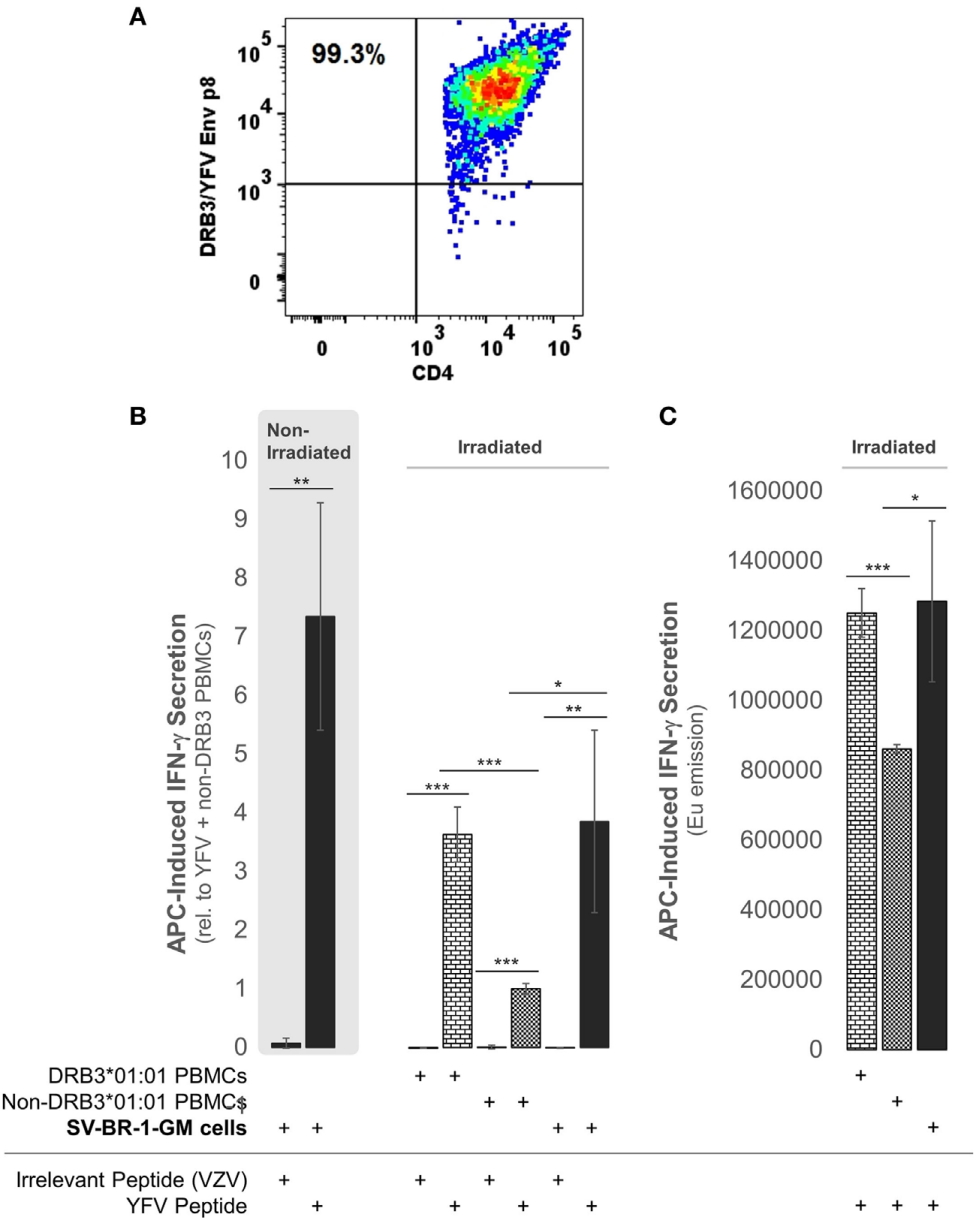
SV-BR-1-GM cells act as antigen-presenting cells (APCs). SV-BR-1-GM cells were cultured and serum-starved for 24 h then coincubated with yellow fever virus (YFV) Envelope (Env) 43–59 peptides (35) known to bind to HLA-DR complexes with an HLA-DRB3*01:01-based β chain and a YFV-DRB3*01:01-specific CD4^+^ T cell clone **(A)**. **(A)** T cell clone after staining with YFV Env p8/DRB3*01:01 tetramers, as assessed by flow cytometry. Almost all T cells are both YFV Env p8/DRB3 and CD4 positive. **(B)** After 72 h of coculturing, T cell activation was assessed by determining the levels of secreted interferon (IFN)-γ. Values shown are arithmetic means from technical triplicates ± SDs, normalized to the mean IFN-γ level obtained from the YFV peptide-treated non-DRB3 PBMC reference wells. Background IFN-γ levels obtained from T cells treated with peptides in the absence of APCs (SV-BR-1-GM or PBMCs) were subtracted. **(C)** IFN-γ levels without background subtraction and normalization of a part of the experiment represented by panel **(B)**. Values shown are arithmetic means of the Europium emission values at 615 nm from technical triplicates ± SDs. **(B,C)** one-tailed Student’s *t*-tests were employed to assess significance, with * referring to 0.01 ≤ *p* < 0.05, ** to 0.001 ≤ *p* < 0.01, and *** to *p* < 0.001.

To establish whether the expression of the Immune Signature, especially the MHC class II components, translates into APC activity, SV-BR-1-GM cells were treated with yellow fever virus (YFV) Envelope (Env) 43–59 peptides (35) and cocultured with a CD4^+^ T cell clone known to recognize such YFV Env peptides when associated with HLA-DRB3*01:01-based HLA-DR complexes (Figure 9A). Indeed, IFN-γ secretion was significantly higher with SV-BR-1-GM cells treated with the YFV Env peptide compared to those obtained *via* an “irrelevant” peptide from VZV or *via* non-DRB3*01:01 PBMCs treated with the YFV peptide (Figures 9B,C). These results identify SV-BR-1-GM cells as APCs for CD4^+^ T cells. Of note, the T cells employed had been previously activated and were extensively expanded prior to their use in the study, thus not representing naïve T cells. Since SV-BR-1-GM cells do not express *CD80* or *CD86*, encoding ligands for the costimulatory receptor CD28, SV-BR-1-GM is unlikely to activate naïve, i.e., nonprimed T cells. On the other hand, lack of *CD80* (*B7-1*) may predict absence of NK cell-mediated destruction of SV-BR-1-GM (93).

## Discussion

With targeted immunotherapies using allogeneic whole-cell preparations, patients are inoculated with a wide variety of antigens of which some may be TAAs coexpressed in patient tumors. However, whether or not an effective immune response is mounted against such TAAs depends on numerous factors. In this report, we present four main lines of evidence suggesting that SV-BR-1-GM cells can act as APCs and thereby could potentially mount an effective tumor-directed immune response. *First*, despite their presumptive breast epithelial origin, SV-BR-1-GM cells express a set of genes including MHC class I and II components associated with immune cells rather than with epithelial cells. *Second*, in a pilot study, a robust clinical response occurred in a clinical trial subject with an *HLA-DRB3* allele match to SV-BR-1-GM. This raises the possibility that in this patient, TAA-MHC complexes expressed on the surface of SV-BR-1-GM cells directly stimulated corresponding T cells. However, since in this pilot study both the number of patients (four evaluable patients), and the number of patients with an *HLA-DRB3* allele match to SV-BR-1-GM (one, A002) are low, it is difficult to estimate the significance of such a match. *Third*, peptide-treated SV-BR-1-GM cells activated cocultured peptide-specific CD4^+^ T cells restricted to the SV-BR-1-GM expressed HLA-DRB3 *01:01 allele. *Fourth*, SV-BR-1-GM cells overexpress TAAs including CTAs such as PRAME.

Among four evaluable clinical trial subjects in a pilot study, three with breast and one with ovarian cancer, objective tumor regression following SV-BR-1-GM inoculation was only seen in one patient (16), in this article referred to as A002. In contrast to the other three patients, subject A002 carried an MHC class II allele (HLA-DRB3*02:02) that was also present in SV-BR-1-GM (Table 2). Additionally, it is worth mentioning that in an ongoing phase I/IIa clinical trial (ClinicalTrials.gov identifier: NCT03066947) similarly conducted as the pilot study, the HLA matching hypothesis found continued support as demonstrated by the regression of multiple pulmonary nodules in a patient with metastatic breast cancer with a mixed clinical response (progression of metastatic disease in the liver) who matched at HLA-A*24:02 and HLA-DRB3*02:02 with SV-BR-1-GM (data not shown).

Together with a set of other genes with known immunostimulatory roles, SV-BR-1-GM cells express a 22-gene Immune Signature (Table 1). From a mechanistic perspective, these findings are consistent with a model in which TAAs are displayed on SV-BR-1-GM cell surface MHCs where they can directly and/or indirectly (upon “cross-dressing,” i.e., upon trogocytosis-based transfer onto APCs such as DCs) activate T cells (Figures S9A,B in Supplementary Presentation S1 in Supplementary Material) (94–96). It should be noted that although while an MHC class II allele match between patient A002 and SV-BR-1-GM may have been key in eliciting an effective anti-tumor immune response, one could envision that patients matching at an expressed MHC class I HLA allele may also benefit from direct antigen presentation provided their tumors express TAAs coexpressed in SV-BR-1-GM. However, even though a study suggested that B7-1 (CD80) positive tumor vaccines may result in some degree of direct antigen presentation to CD8^+^ T cells (97), in a mouse model, MHC class I-restricted tumor antigens were not detectably presented by the tumor itself (98), thus questioning whether MHC class I expression by SV-BR-1-GM cells indeed contributes to the cell line’s therapeutic potential. Furthermore, it is important to emphasize that the data presented here does not exclude cross-presentation, the more classical mechanism for tumor antigen display, as an additional contributor to SV-BR-1-GM’s MoA. It even seems very reasonable to hypothesize that after direct T cell stimulation by live, irradiated SV-BR-1-GM cells, antigens from apoptotic SV-BR-1-GM cells are taken up by DCs and used to activate patient T cells (Figure S9C in Supplementary Presentation S1 in Supplementary Material). With such a mechanism, targeted immunotherapy with SV-BR-1-GM would both directly and indirectly stimulate patient T cells, with only the former requiring HLA matching between SV-BR-1-GM and the patient.

Since the proposed MoA of the SV-BR-1-GM regimen (Figure S9A in Supplementary Presentation S1 in Supplementary Material) may similarly apply to other GVAX immunotherapies, one may wonder whether in such programs patients with MHC class I and/or II allele matches had better clinical responses to the vaccine than those without. Moreover, if HLA alleles indeed contribute to the efficacy of GVAX immunotherapies, high-resolution HLA typing should be considered as a companion diagnostic. Table 5 outlines estimated “phenotype frequencies” (PFs) of HLA-A, -B, and -DRB3 alleles expressed in SV-BR-1-GM for different subpopulations. As shown, combined HLA-DRB3*01:01 &*02:02 PFs range from 29.7 to 68.2%, i.e., the probability that a randomly selected individual carries at least one of SV-BR-1-GM’s expressed HLA-DRB3 alleles is 29.7–68.2% depending on the subpopulation. When loosening restrictions to only consider the allele groups, combination frequencies are only marginally higher (ranging from 29.8 to 68.6%) since SV-BR-1-GM’s HLA-DRB3 alleles (*01:01 and *02:02) are the most frequent alleles of the DRB3*01 and *02 allele groups (36).

**Table 5:** “Phenotype Frequencies” of HLA alleles expressed in SV-BR-1-GM cells.

HLA allele matches with SV-BR-1-GM		AAFA	AFB	AINDI	AMIND	CARB	CARHIS	EURCAU	FILII	JAPI	KORI	MENAFC	MSWHIS	NCHI	SCAHIS	SCSEAI	VIET
Accuracy	Per individual																
Allele group	≥1 HLA-A	5.1	4.6	29.8	25.3	5.2	19.1	16.6	64.4	58.9	39.6	23.8	25.2	29.7	25.8	32.1	29.7
	≥1 HLA-B	14.8	14.4	30.3	24.3	16.4	22.5	20.0	22.8	22.2	16.0	31.1	30.6	12.8	32.1	26.1	11.9
	≥1 HLA-DRB3	64.7	63.7	54.1	53.9	63.1	55.7	55.2	29.8	30.7	40.1	68.6	52.1	46.1	52.0	52.3	31.1

Allele	≥1 HLA-A	4.8	4.4	25.4	24.2	5.0	17.9	16.2	42.2	58.1	39.3	22.1	24.4	28.1	24.6	25.7	21.0
	≥1 HLA-B	0.9	0.7	4.4	3.1	0.9	2.6	4.5	0.3	0.2	0.2	7.8	2.8	0.3	3.0	3.6	0.2
	≥1 HLA-DRB3	64.6	63.7	54.1	53.8	63.0	55.6	55.2	29.7	30.6	40.1	68.2	52.1	46.1	51.9	52.1	31.0

From the allele frequencies reported by Gragert et al (36)., estimated “phenotype frequencies” were calculated indicating probabilities that an individual carries at least 1 of SV-BR-1-GM’s expressed HLA-A, HLA-B, or HLA-DRB3 alleles (HLA-A*24:02, HLA-B*35:08, HLA-B*55:01, HLA-DRB3*01:01, HLA-DRB3*02:02) or allele groups (HLA-A*24, HLA-B*35, HLA-B*55, HLA-DRB3*01, HLA-DRB3*02). Shown are “phenotype frequencies” as percentage values. See Section “Materials and Methods” for details on the calculations. AAFA, African American; AFB, African; AINDI, South Asian Indian; AMIND, North American Indian; CARB, Caribbean black; CARHIS, Caribbean Hispanic; EURCAU, European Caucasian, FILII, Filipino; JAPI, Japanese; KORI, Korean; MENAFC, Middle Eastern or N. Coast of Africa; MSWHIS, Mexican or Chicano; NCHI, Chinese; SCAHIS, Hispanic—South or Central American; SCSEAI, Southeast Asian; VIET, Vietnamese. Since HLA-A*11:01 may not be expressed in SV-BR-1-GM cells (Figure 3A), only HLA-A frequency values for A*24/A*24:02 were used. Similarly, HLA-C frequencies were not included as HLA-C RNA was not expressed at levels considered relevant (data not shown).

To mitigate the risk for tumor development by the vaccine itself, SV-BR-1-GM cells are irradiated with 200 Gy (20,000 rad) prior to their clinical application (16). Interestingly, Sharma et al. demonstrated that *ex vivo* gamma-irradiation may upregulate both MHC class I and CTAs in cancer cell lines representing different cancer types and in biopsy samples from sarcoma patients. Importantly, such gene expression changes were accompanied by increased recognition by CD8^+^ cells (99). This notion is also important clinically, as there is evidence suggesting that tumor irradiation could enhance the benefits of immunotherapy (100–102). Surprisingly, we observed *reduced* HLA-DR cell surface levels on irradiated compared to nonirradiated SV-BR-1-GM cells (Figure 5). However, it is unclear whether for SV-BR-1-GM cells, irradiation with 200 Gy indeed downregulates HLA-DR cell surface expression or whether differences in the handling of the cell preparations have accounted for the observed results. Furthermore, HLA-DR expression was heterogeneous, with only some 15% of the irradiated SV-BR-1-GM cells staining positive for HLA-DR. However, since each treatment cycle under the current protocol includes inoculation of 20 million SV-BR-1-GM cells, distributed into four intradermal sites (5 million cells per site), each patient is still believed to receive enough HLA-DR^+^ cells to induce the postulated HLA-dependent immune response.

It can be hypothesized that, in addition to matching HLA alleles, TAAs coexpressed in the vaccine and patient tumors were chiefly responsible for the favorable course of action observed in patient A002. In the molecular study presented here we sought to identify candidate TAAs whose overexpression in SV-BR-1-GM cells may break immunologic tolerance.

Immunologic tolerance is a double-edged sword. Its underlying mechanisms prevent both autologous antigens from evoking an immune response (autoimmunity) and the recognition of tumors by the immune system. Several methods to break tolerance have been described, including the use of immune checkpoint inhibitors or monoclonal antibodies delivering costimulatory signals to T cells (103). In the context of targeted cancer immunotherapy with live, irradiated whole-cell preparations, GM-CSF secreted by the cell line has been attributed a major role in overcoming immune tolerance (9). However, given that GM-CSF-expressing whole-cell vaccines express a vast array of antigens coexpressed in healthy cells, one would imagine that autoimmunity may accompany such treatments. Indeed, thyroglobulin antibody seroconversion following GVAX immunotherapy has been reported (and was associated with prolonged survival). However, since thyroglobulin was not found to be expressed in the GVAX cell lines used, the development of the thyroglobulin antibodies was likely based on alternative mechanism(s) (20).

We hypothesized that TAAs responsible for SV-BR-1-GM’s anti-tumor effect might be overexpressed in the cell line and as such could mediate the breakage of immune tolerance. This was addressed by means of a 2-tier microarray-based *in silico* approach. First, we wanted to identify genes upregulated in SV-BR-1-GM cells relative to normal breast cells. This was accomplished by comparing several different lots of SV-BR-1-GM to a variety of normal human breast cell types. Second, the genes with apparently higher expression levels in SV-BR-1-GM compared with normal breast cells were subjected to an *in silico* verification step for which the genes’ expression levels in breast cancer were compared to those of normal tissues of various organs. Since breakage of immune tolerance by overexpression may, at least in principle, only occur for genes with no/low physiological expression at every site permissive for immune surveillance, we reasoned that ideal candidate immunogens should be highly expressed in SV-BR-1-GM cells and breast cancer tissues but not, or only minimally, in normal tissues other than immune-privileged sites. The bioinformatics strategy applied to verify immunogen candidates reflects this theory. Thirty-one genes encoding candidate TAAs were considered more highly expressed in both SV-BR-1-GM cells and breast cancer tissues than in normal tissues (Table 4). Interestingly, among these thirty-one genes, six were located on chromosome 17 of which four mapped to 17q12, namely *ERBB2* (*HER2*/*neu*), *MIEN1* (*C17orf37*), *PGAP3* (*PERLD1*), an*d STARD3* (Figure S8 in Supplementary Presentation S1 in Supplementary Material). This suggests that not only *ERBB2* may be amplified in a subset of breast cancers but also other candidate TAAs located in vicinity of *ERBB2* may be coamplified in the same cells (104, 105). Interestingly, in contrast to *ERBB2*, relatively little is known about *MIEN1* (106–108) and *PGAP3* (109), thus providing opportunities for further exploration. High StARD3 protein levels with a strong association with *HER2* amplification was reported for approximately 10% of breast cancers in two Finnish nationwide patient cohorts (110).

Cancer/testis antigens are a class of TAAs specifically expressed in cancer and germline tissues (6, 32–34, 88–91). The stringent filtering approach which yielded 31 *in silico* verified TAA candidates did not select for CTAs. However, when gene expression profiles of a set of 279 CTAs (Data Sheets S4 in Supplementary Material) were analyzed, several CTAs, most notably *PRAME*, were found to be selectively expressed in SV-BR-1-GM compared to normal breast cells (Figures 7 and 8; Table 3). Even though it was not found to be expressed in noncancerous tissues other than tissues of the testis and the endometrium, *PRAME* was missed in the stringent *in silico* screen because in the 54 breast cancer specimens analyzed, *PRAME* expression was restricted to only 11 (20%) samples of which only 2 (4%) demonstrated appreciable expression levels (data not shown). Furthermore, at least some of the CTAs may have low expression levels in SV-BR-1-GM cells (Figures 7 and 8). However, as demonstrated by Groeper et al., CTA-specific TILs could even be expanded from tumors with undetectable CTA levels (6). This may suggest that minuscule (below level of detection) CTA expression levels may suffice for CTA-specific T cell retention in the tumor or that such T cells only coincidentally happened to reside in the tumor tissue as it was resected. In agreement with the latter possibility, CTA-directed cytotoxicity of TILs from tumors with undetectable CTA expression was weak (6).

In summary, the study presented here supports a model in which SV-BR-1-GM’s tumor-directed effects reported previously (16) were mediated at least in part by the cell line’s 22-gene “Immune Signature,” which includes factors ranging from MHC class I and II components to ligands for T cell costimulatory receptors and chemokines known to promote attraction of immune cells, and by TAAs such as PRAME. Importantly, peptide-treated SV-BR-1-GM cells selectively activated pMHC-specific CD4^+^ T cells, which confirms that SV-BR-1-GM cells can act as APCs and suggests that this functionality is critical for SV-BR-1-GM’s MoA.

## Conclusion

Unlike other established breast cancer cell lines, SV-BR-1-GM cells not only express known and putative TAAs; they also express a collection of factors with known roles in promoting immune responses. Most notably, in addition to MHC class I factors, also class II genes such as *HLA-DMA, -DMB, -DRA*, and *-DRB3* are expressed. Since MHC class II components are associated with *bone fide* APCs such as DCs, their expression in SV-BR-1-GM cells is surprising and may point to a unique MoA. Since the patient who responded to the SV-BR-1-GM regimen with tumor regression (16) carried an MHC class II allele also expressed in SV-BR-1-GM cells, we hypothesize that patients coexpressing SV-BR-1-GM TAAs *and* expressing matching HLA alleles are more likely to develop a strong tumor-directed immune response than those without such characteristics.

## Data Availability

Microarray data of the 22 samples passing QC (i.e., excluding CP Lot V cryo) discussed in this publication have been deposited in NCBI’s Gene Expression Omnibus (28) and are accessible through GEO Series accession number GSE112239 (https://www.ncbi.nlm.nih.gov/geo/query/acc.cgi?acc=GSE112239).

## Ethics Statement

The clinical aspect of this study was conducted with US Food and Drug Administration (FDA) and St. Vincent Medical Center institutional review board (IRB) approval, and written informed patient consent was obtained (16). The clinical trial was registered under ClinicalTrials.gov Identifier NCT00095862.

## Author Contributions

ML conceived the study and interpreted data, conducted bioinformatics analyses and part of the wet laboratory experiments, and wrote the manuscript. GB lead the manufacturing of CP Lot VIII and oversaw technical procedures. BF and EF expanded MCB cells to CP Lot VIII and conducted experiments. TP and TH expanded cells. DC-B, SG, and YK conducted experiments. WK, JW, and WW provided intellectual input. CW oversaw the manufacturing of several SV-BR-1-GM lots and provided intellectual input. All authors read and approved the final manuscript.

## Conflict of Interest Statement

BriaCell Therapeutics Corp. owns SV-BR-1-GM and the following SV-BR-1-GM-associated intellectual property: (1) US7674456 (issued US patent) and (2) PCT/US2017/019757 (international patent application). Furthermore, BriaCell Therapeutics Corp. acts as Sponsor on two current SV-BR-1-GM clinical trials (ClinicalTrials.gov Identifiers NCT03066947 and NCT03328026). ML, SG, CW, and WW have equity in BriaCell Therapeutics Corp. The other authors declare that they have no competing interests.

## Supplementary Material

Presentation 1 Figure S1: Hierarchical clustering of SV-BR-1-GM samples in comparison to other human breast cancer cell lines (A and B) or normal human breast cells (B). Heat maps of genes regulated similarly as *ERBB2* are shown in C. Figure S2 demonstrates microarray-based gene expression levels of MHC class I and Figure S3 of MHC class II components in SV-BR-1-GM cells. Figure S4: Confirmation of several MHC class II components by quantitative RT-PCR. Figure S5: Levels of GM-CSF secreted by nonirradiated SV-BR-1-GM cells are shown. Figure S6: Overview of the *in silico* filtration strategy to identify candidate TAAs. Figure S7: Illustration of the low- and medium-stringency *in silico* filtration approach. Figure S8: Genes expressed in SV-BR-1-GM cells and located on chromosome 17q12 (“*ERBB2* amplicon”). Figure S9: Hypothetical mechanism of action of SV-BR-1-GM as a therapeutic cancer vaccine (**A**). Factors expressed in SV-BR-1-GM cells and some of their known roles as immune modulators. Expression of MHC class I and II genes is consistent with a model in which SV-BR-1-GM cells directly stimulate cytotoxic T lymphocytes (CD8^+^) and T helper cells (CD4^+^), and thereby, potentially, induce both cytotoxic and humoral responses. The presence of functional MHC class II is unexpected given the cells’ presumptive breast epithelial origin and may in part be responsible for the tumor-directed clinical effects observed in patients matching at an HLA class II allele with SV-BR-1-GM. Nevertheless, since SV-BR-1-GM cells do not express *CD80* or *CD86* mRNA they unlikely act directly as antigen-presenting cells activating naïve T cells. However, activation of naïve T cells may occur *via* dendritic cells (DCs), after direct transfer of tumor-associated antigen (TAA)-MHC complexes from the cell surface of SV-BR-1-GM cells to the cell surface of DCs by means of trocycytosis (cross-dressing) (**B**) and/or by uptake and intracellular processing of SV-BR-1-GM antigens *via* cross-presentation (**C**). CTL, cytotoxic T lymphocyte; T_H_, T helper cell. Shown is a subset of the factors with immunomodulatory roles expressed in SV-BR-1-GM cells. Additional factors are listed in Table 1.

Data Sheet 1 Accession numbers and descriptions of normal tissue samples from GEO DataSet GSE7307 used for the *in silico* verification step of candidate TAAs are shown.

Data Sheet 2 Reagents and samples for quantitative RT-PCR and nCounter-based verification of gene expression are shown.

Data Sheet 3 List of genes with immunostimulatory roles and Immune Signature candidates are shown.

Data Sheet 4 A list of cancer/testis antigens (CTAs) is provided.

Data Sheet 5 Genes retained after the low- and medium *in silico* filtration steps are shown.

## Abbreviations

APC, antigen-presenting cell; CTA, cancer/testis antigen; DC, dendritic cell; HGNC, Human Genome Organisation (HUGO) Gene Nomenclature Committee; HLA, human leukocyte antigen; HMEC, human mammary epithelial cell; MHC, major histocompatibility complex; MoA, mechanism of action; pMHC, peptide-loaded MHC; qRT-PCR, quantitative reverse transcription-polymerase chain reaction; TAA, tumor-associated antigen.
