# Supplementary material for: SV-BR-1-GM, a Clinically Effective GM-CSF-Secreting Breast Cancer Cell Line, Expresses an Immune Signature and Directly Activates CD4+ T Lymphocytes
Source: Front Immunol. 2018 May 15;9:776. doi: 10.3389/fimmu.2018.00776 (PMC5962696; doi:10.3389/fimmu.2018.00776)
Supplement: Presentation 1 — Figure S1: Hierarchical clustering of SV-BR-1-GM samples in comparison to other human breast cancer cell lines (A and B) or normal human breast cells (B). Heat maps of genes regulated similarly as ERBB2 are shown in C. Figure S2 demonstrates microarray-based gene expression levels of MHC class I and Figure S3 of MHC class II components in SV-BR-1-GM cells. Figure S4: Confirmation of several MHC class II components by quantitative RT-PCR. Figure S5: Levels of GM-CSF secreted by nonirradiated SV-BR-1-GM cells are shown. Figure S6: Overview of the in silico filtration strategy to identify candidate TAAs. Figure S7: Illustration of the low- and medium-stringency in silico filtration approach. Figure S8: Genes expressed in SV-BR-1-GM cells and located on chromosome 17q12 (“ERBB2 amplicon”). Figure S9: Hypothetical mechanism of action of SV-BR-1-GM as a therapeutic cancer vaccine (A). Factors expressed in SV-BR-1-GM cells and some of their known roles as immune modulators. Expression of MHC class I and II genes is consistent with a model in which SV-BR-1-GM cells directly stimulate cytotoxic T lymphocytes (CD8+) and T helper cells (CD4+), and thereby, potentially, induce both cytotoxic and humoral responses. The presence of functional MHC class II is unexpected given the cells’ presumptive breast epithelial origin and may in part be responsible for the tumor-directed clinical effects observed in patients matching at an HLA class II allele with SV-BR-1-GM. Nevertheless, since SV-BR-1-GM cells do not express CD80 or CD86 mRNA they unlikely act directly as antigen-presenting cells activating naïve T cells. However, activation of naïve T cells may occur via dendritic cells (DCs), after direct transfer of tumor-associated antigen (TAA)-MHC complexes from the cell surface of SV-BR-1-GM cells to the cell surface of DCs by means of trocycytosis (cross-dressing) (B) and/or by uptake and intracellular processing of SV-BR-1-GM antigens via cross-presentation (C). CTL, cytotoxic T ly [file Presentation_1.PDF]

SV-BR-1-GM, a Clinically Effective GM-CSF-Secreting Breast Cancer Cell Line, Expresses an Immune Signature and Directly Activates CD4+ T Lymphocytes

Markus D. Lacher, Gerhard Bauer, Brian Fury, Sanne Graeve, Emily L. Fledderman, Tye D. Petrie, Dane P. Coleal-Bergum, Tia Hackett, Nicholas H. Perotti, Ying Y. Kong, William W. Kwok, Joseph P. Wagner, Charles L. Wiseman, and William V. Williams

---

Supplementary Presentation 1

A

SV-BR-1-GM vs. Other Established Breast Cell Lines

FIG. S1

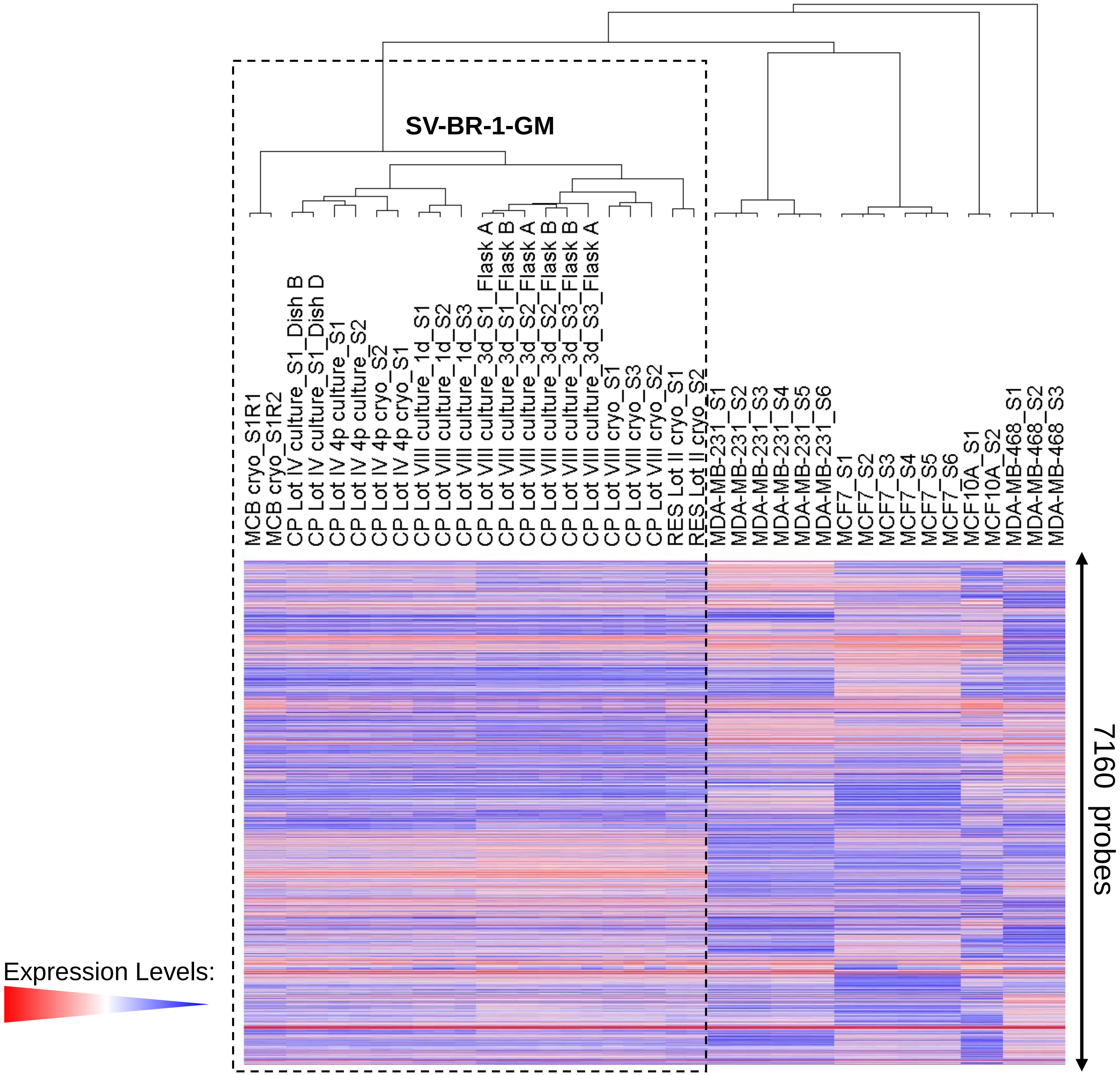

B

FIG. S1

SV-BR-1-GM vs. Other Breast Cancer and Normal Breast Cells

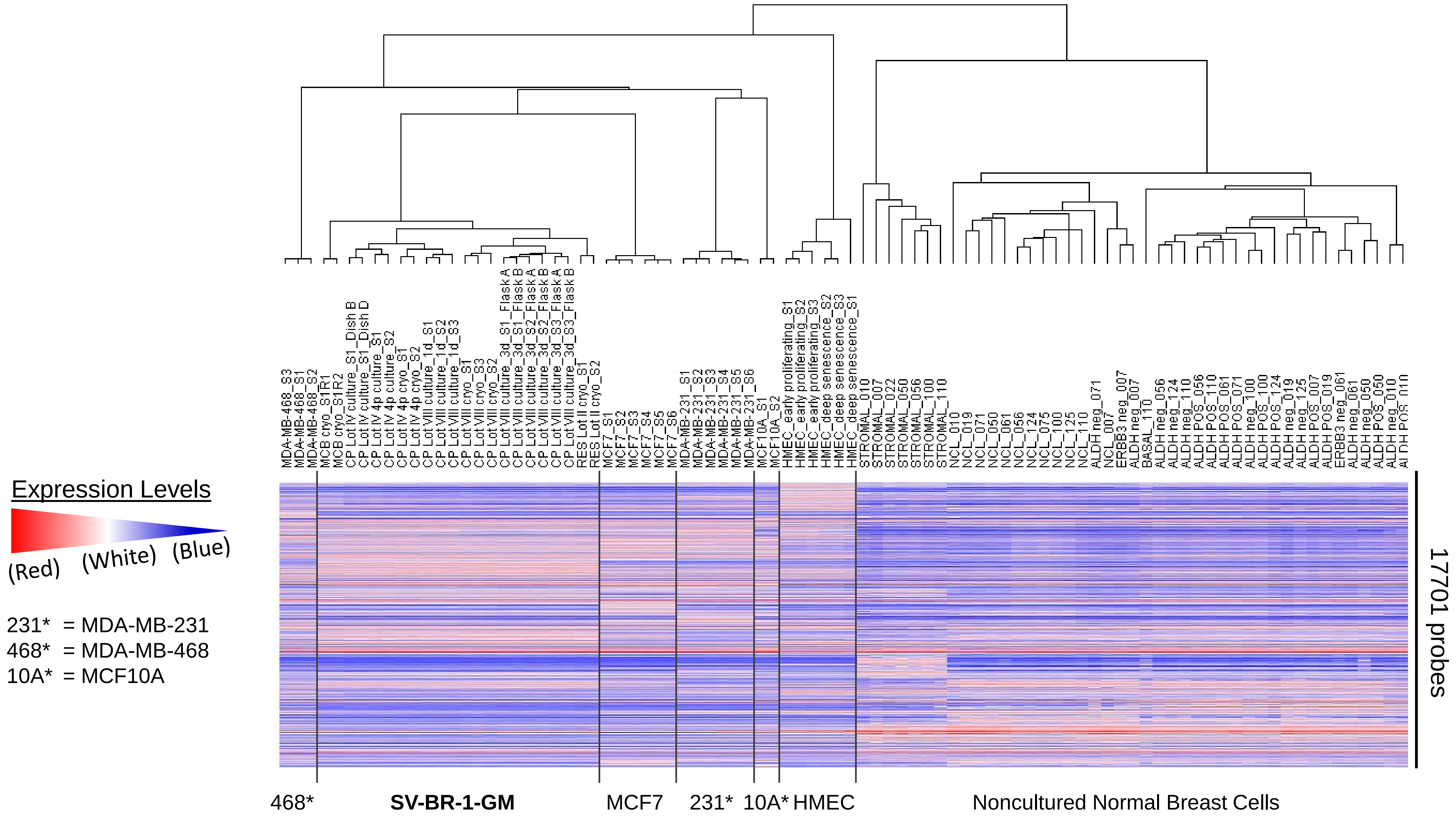

C

FIG. S1

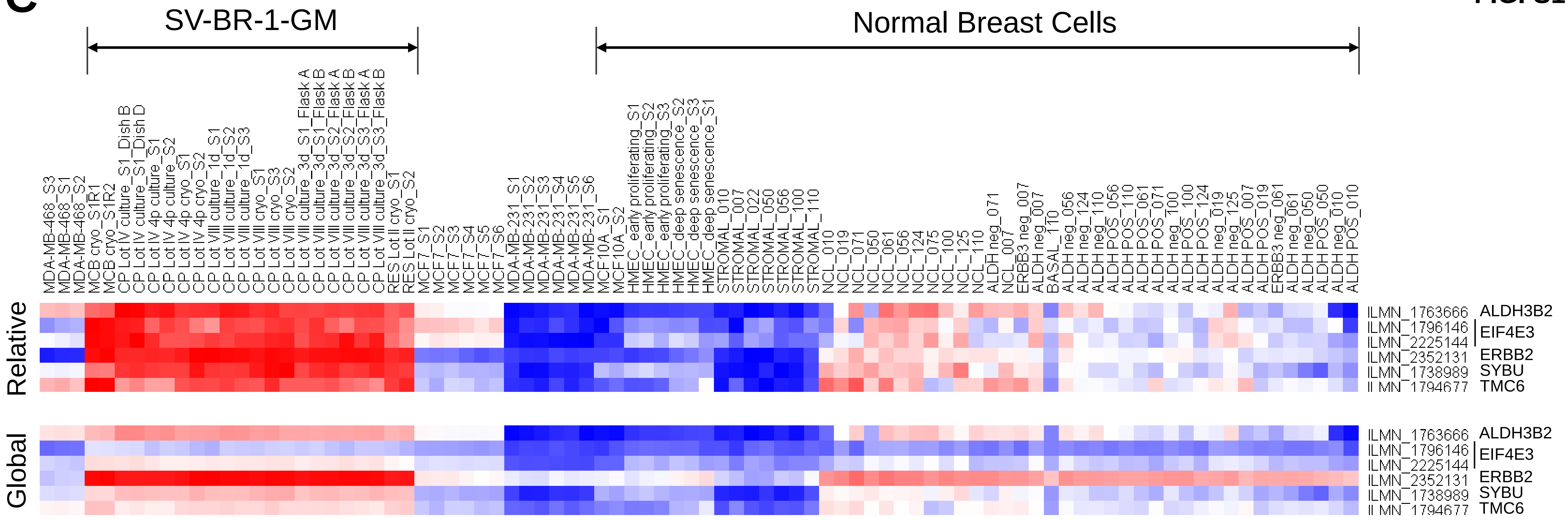

**Gene Symbol (NCBI)**	**Official Full Name (NCBI)**	**Chr. Location**
ALDH3B2	aldehyde dehydrogenase 3 family member B2	11q13.2
EIF4E3	eukaryotic translation initiation factor 4E family member 3	3p13
ERBB2	erb-b2 receptor tyrosine kinase 2	17q12
SYBU	syntabulin	8q23.2
TMC6	transmembrane channel like 6	17q25.3

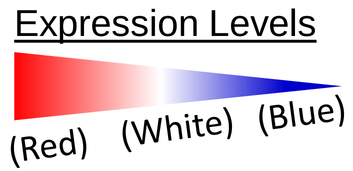

Legend to Figure S1

A. **Hierarchical Clustering** of both samples and genes (probes) with minimum expression values among all samples > 1.5 times the background cutoff value. The SV-BR-1-GM samples cluster separately from the MDA-MB-231, MDA-MB-468, MCF7, and MCF10A samples.

B. **Hierarchical Clustering** of both samples and genes (probes) with maximum expression values among the different sample groups (SV-BR-1-GM, MDA-MB-231, MDA-MB-468, MCF7, MCF10A , ALDH NEG, ALDH POS, ERBB3 NEG, NCL, BASAL, STROMAL, HMEC_early proliferating, HMEC_deep senescence) > 1.5 times the background cutoff value. Cell lines and noncultured breast cells build separate clusters; the SV-BR-1-GM samples build their own subcluster within the cell line group.

C. ***ERBB2* Cluster.** *ALDH3B2*, *EIF4E3*, *SYBU*, and *TMC6* cluster tightly with *ERBB2* across the samples indicated. “Global” vs. “Relative” refers to heat map coloring based on all of the expression values represented (“Global”) or based on only those of the corresponding gene, i.e., row (“Relative”). As evidenced from the “Global” display, *ERBB2*, in both SV-BR-1-GM and normal breast cells, is expressed at higher levels than the other genes. Chr. Location: chromosomal location as indicated on the respective NCBI Gene sites.

MCF7, MDA-MB-231, and MDA-MB-468 are human breast cancer cell lines. MCF10A is a “normal” human epithelial cell line. HMEC, human mammary epithelial cells. Data sets other than of SV-BR-1-GM were obtained from GEO (NCBI) and are as follows: noncultured breast cells from GSE35399 (Shehata et al., *Breast Cancer Res.* 2012;14(5):R134), HMECs from GSE56718 (Lowe et al., *Genome Biol.* 2015 Sep 17;16:194), and MCF7, MCF10A, MDA-MB-231, and MDA-MB-468 from GSE48398.

HLA Class I Components in SV-BR-1-GM by Microarray

FIG. S2

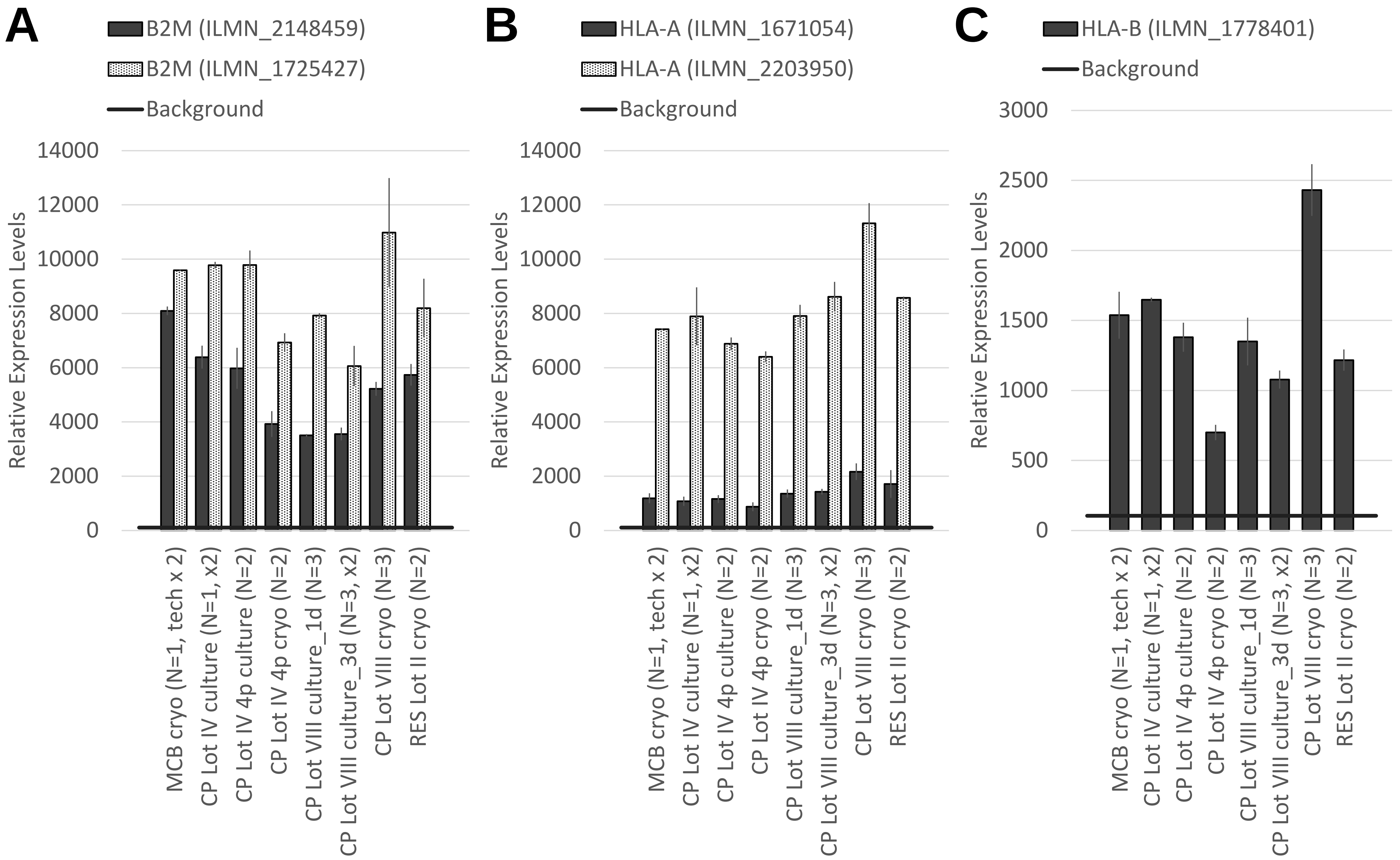

FIG. S2

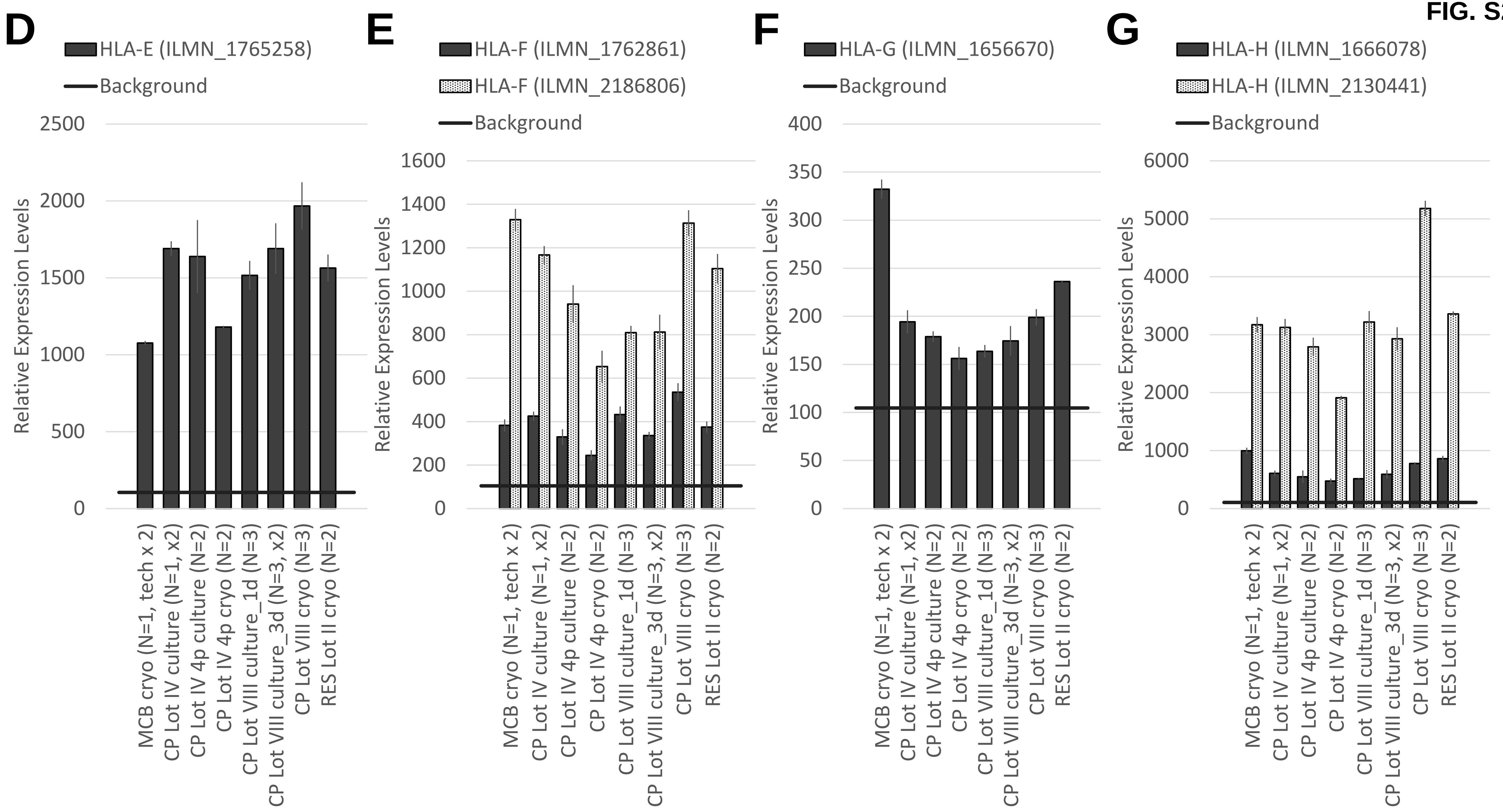

D
HLA-E (ILMN_1765258)
Background
Relative Expression Levels
2500
2000
1500
1000
500
0
MCB cryo (N=1, tech x 2)
CP Lot IV culture (N=1, x2)
CP Lot IV 4p culture (N=2)
CP Lot IV 4p cryo (N=2)
CP Lot VIII culture_1d (N=3)
CP Lot VIII culture_3d (N=3, x2)
CP Lot VIII cryo (N=3)
RES Lot II cryo (N=2)
E
HLA-F (ILMN_1762861)
HLA-F (ILMN_2186806)
Background
Relative Expression Levels
1600
1400
1200
1000
800
600
400
200
0
F
HLA-G (ILMN_1656670)
Background
Relative Expression Levels
400
350
300
250
200
150
100
50
0
G
HLA-H (ILMN_1666078)
HLA-H (ILMN_2130441)
Background
Relative Expression Levels
6000
5000
4000
3000
2000
1000
0

Legend to Figure S2

SV-BR-1-GM expresses both “classical” HLA-Ia and “nonclassical” HLA-Ib components. “Relative Expression Levels” refers to quantile-normalized mRNA levels obtained via microarray hybridization. **A.** *B2M*, encoding β2-microglobulin, **B.** *HLA-A*, **C.** *HLA-B*, **D.** *HLA-E*, **E.** *HLA-**F***, **F.** *HLA-G*, and **G.** *HLA-H*.

HLA Class II Components by Microarray

FIG. S3

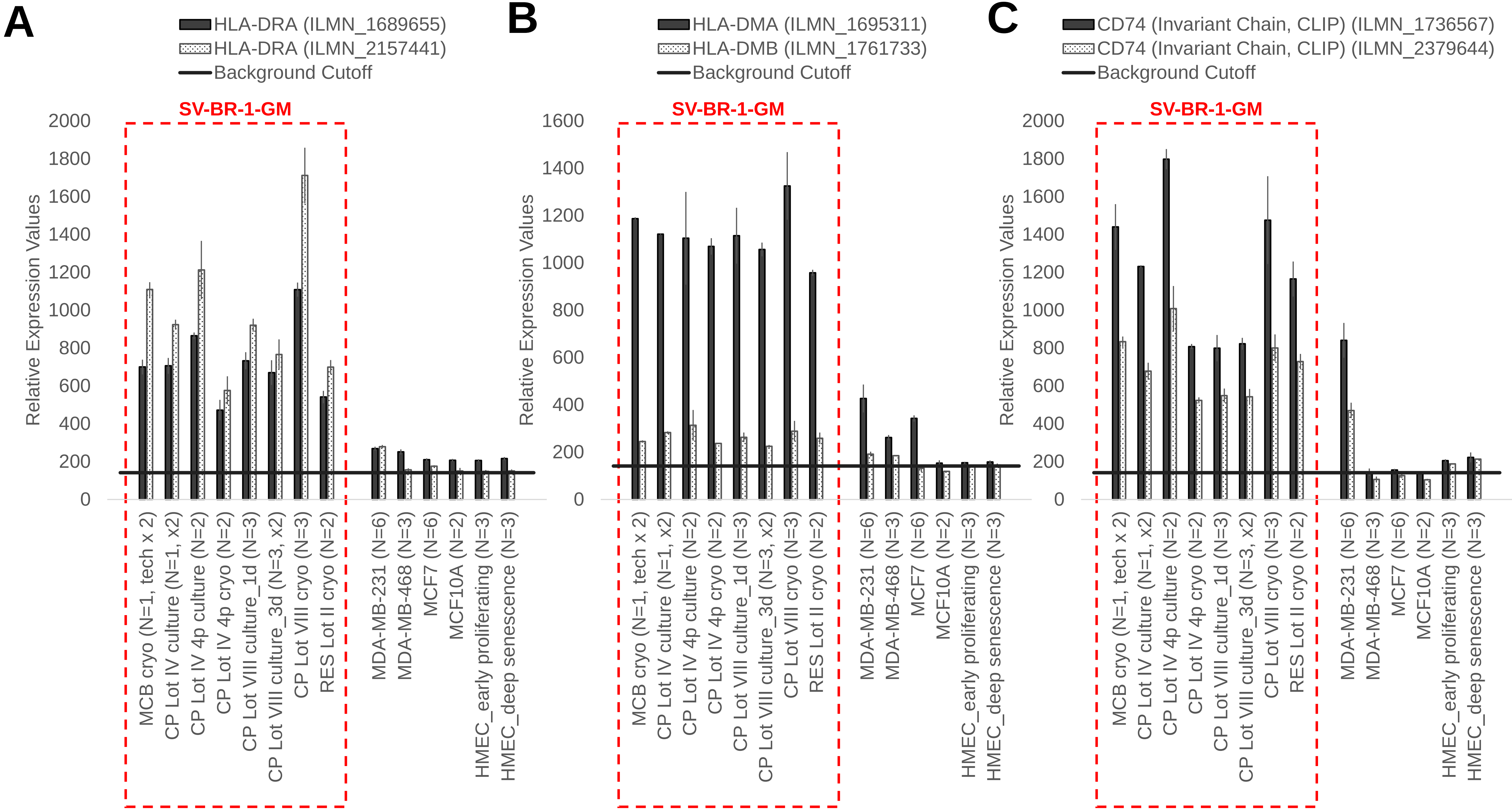

FIG. S3

Legend to Figure S3

HLA class II components in SV-BR-1-GM cells. SV-BR-1-GM cells express components predictive for functional HLA-DR complex formation. “Relative Expression Values” refers to quantile-normalized mRNA levels obtained via microarray hybridization**.** **(A)** *HLA-DRA*, encoding an HLA-DR alpha chain, **(B)** *HLA-DMA* and *HLA-DMB*, encoding HLA-DM, a non-classical MHC II which chaperones peptide-free MHC II against inactivation and catalyzes the exchange of the CLIP peptide with peptides from endocytosed or endogenous antigens (Guce et al., *Nat Struct Mol Biol.* 2013 Jan;20(1):90-8), **(C)** *CD74*, encoding Invariant Chain and CLIP.

HLA Class II Components by Quantitative RT-PCR

FIG. S4

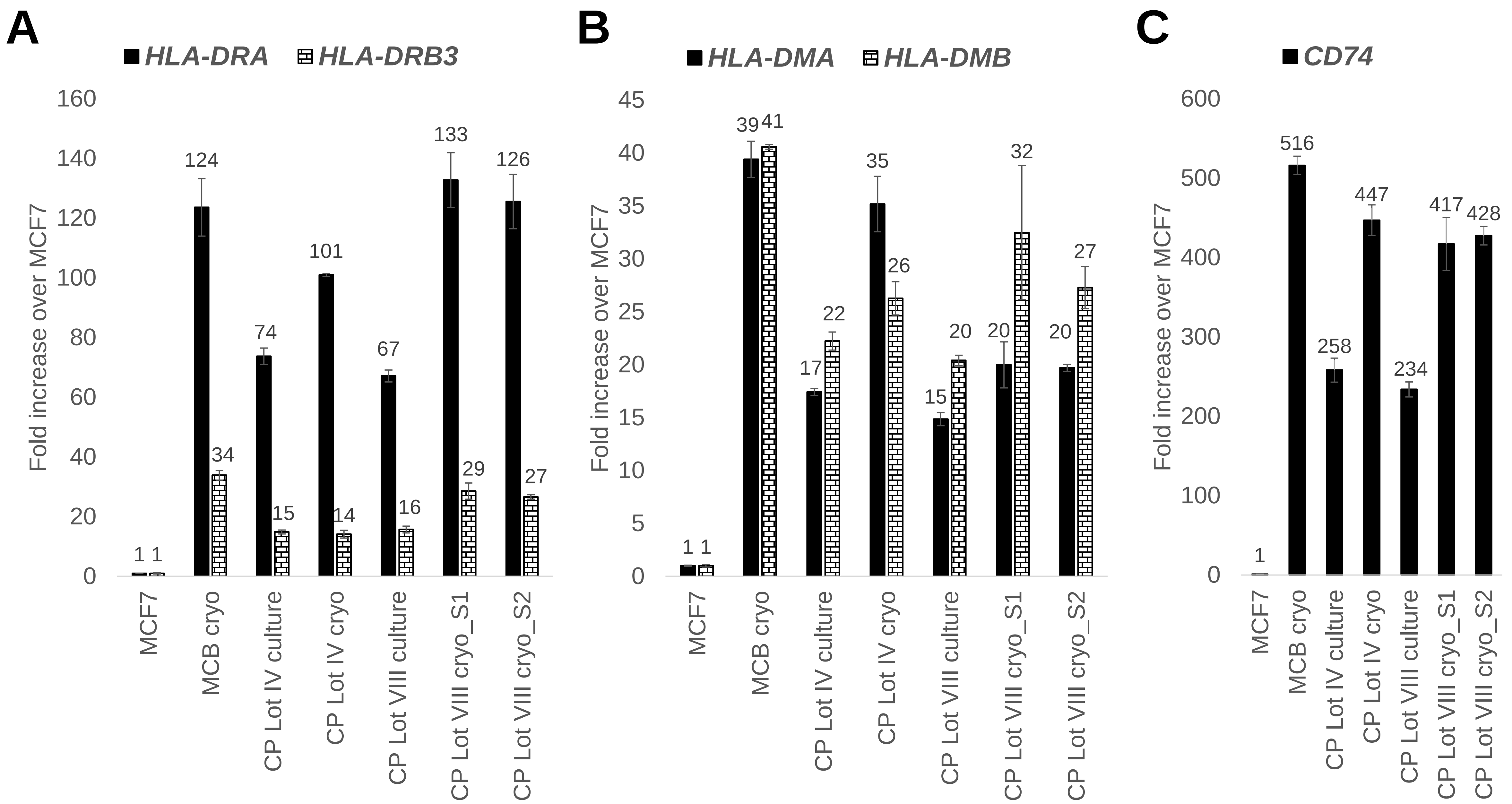

FIG. S4

Legend to Figure S4

Verification of HLA class II gene expression by quantitative RT-PCR. To verify the expression of several critical HLA class II components a confirmatory experiment was conducted on a subset of the SV-BR-1-GM samples (**Table S2 in Supplementary Data Sheet 2**) used for Illumina microarray analysis and with RNA from MCF7 cells [breast cancer cell line carrying the *HLA-DRB3*0202* allele (Edgecombe AD. HLA class II expression on breast cancer cells (Ph.D. Thesis). Faculty of Medicine, Memorial University of Newfoundland, Canada. URL: http://research.library.mun.ca/9160/. (2002))] as calibrator sample. All MHC II-related transcripts analyzed, **(A)** *HLA-DRA* and *HLA-DRB3*, **(B)** *HLA-DMA* and *HLA-DMB*, and **(C)** *CD74*, were expressed in SV-BR-1-GM cells at substantially higher levels than in MCF7 cells.

FIG. S5

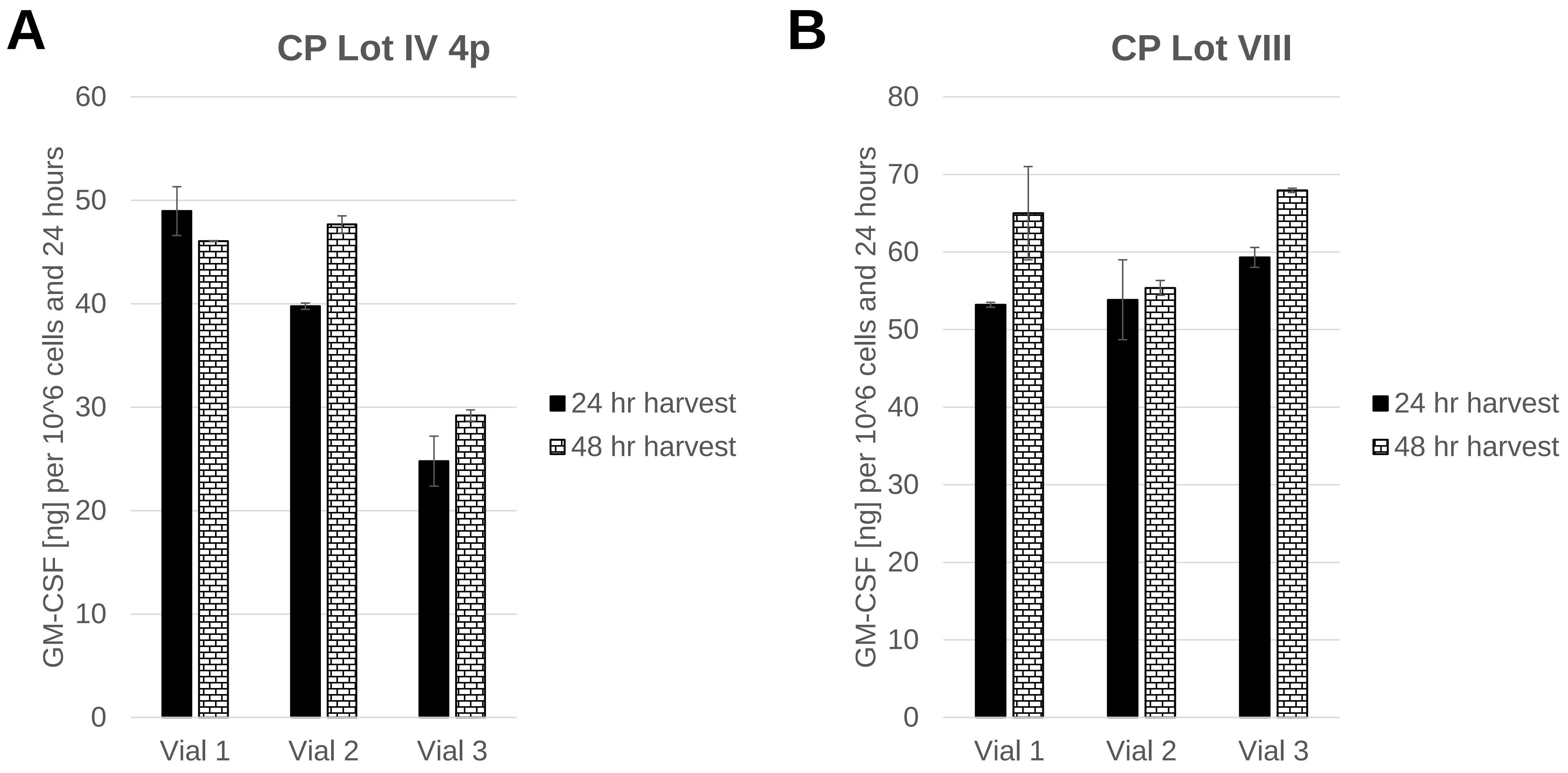

GM-CSF secretion by nonirradiated SV-BR-1-GM cells. For each sample type, SV-BR-1-GM CP Lot IV 4p (**A**) and SV-BR-1-GM CP Lot VIII (**B**), GM-CSF production from cells obtained from three (3) cryovials (vials 1-3) was measured. From each cryovial, cells were seeded into three (3) T-75 flasks (~4 million cells/flask). 2 days later (t = 0 hours), the culture media from two (2) flasks per cryovial were replaced with 14 ml/flask of full medium and the cells from the third flask enumerated and harvested (1st harvest day, yielding RNA for microarray). 24 and 48 hours after the media change, aliquots of the culture supernatants were harvested and cryopreserved. 48 hours after the media change, also cells were harvested (3rd harvest day, yielding RNA for microarray). GM-CSF secretion was assessed from the culture supernatants by ELISA (Human GM-CSF Quantikine ELISA Kit; R&D Systems/bio-techne, Minneapolis, MN). Data is expressed as ng GM-CSF per 1 million cells and 24 hours (relative to cell numbers at t = 0 hours).

FIG. S6

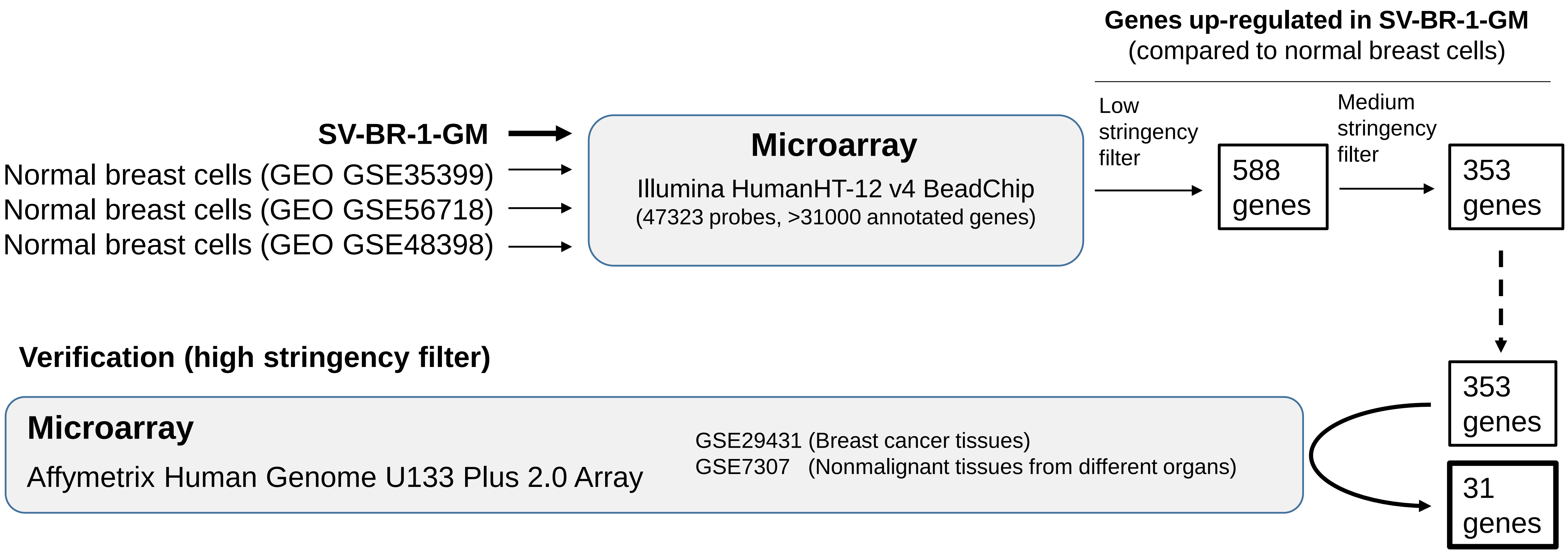

Overview of the filtration strategy to identify candidate TAAs. Gene expression profiles of SV-BR-1-GM cells were compared to those of normal breast cells (subset of samples represented by GSE35399, GSE56718, GSE48398). 588 genes (NCBI Gene Symbols) were retained after applying a low stringency filter; 353 of them were also retained in the medium stringency filter. These 353 genes were then subjected to an *in silico* verification step aimed at identifying genes that are overexpressed both in SV-BR-1-GM cells and breast cancer tissue, but lack expression in nonmalignant tissues of various organs.

FIG. S7

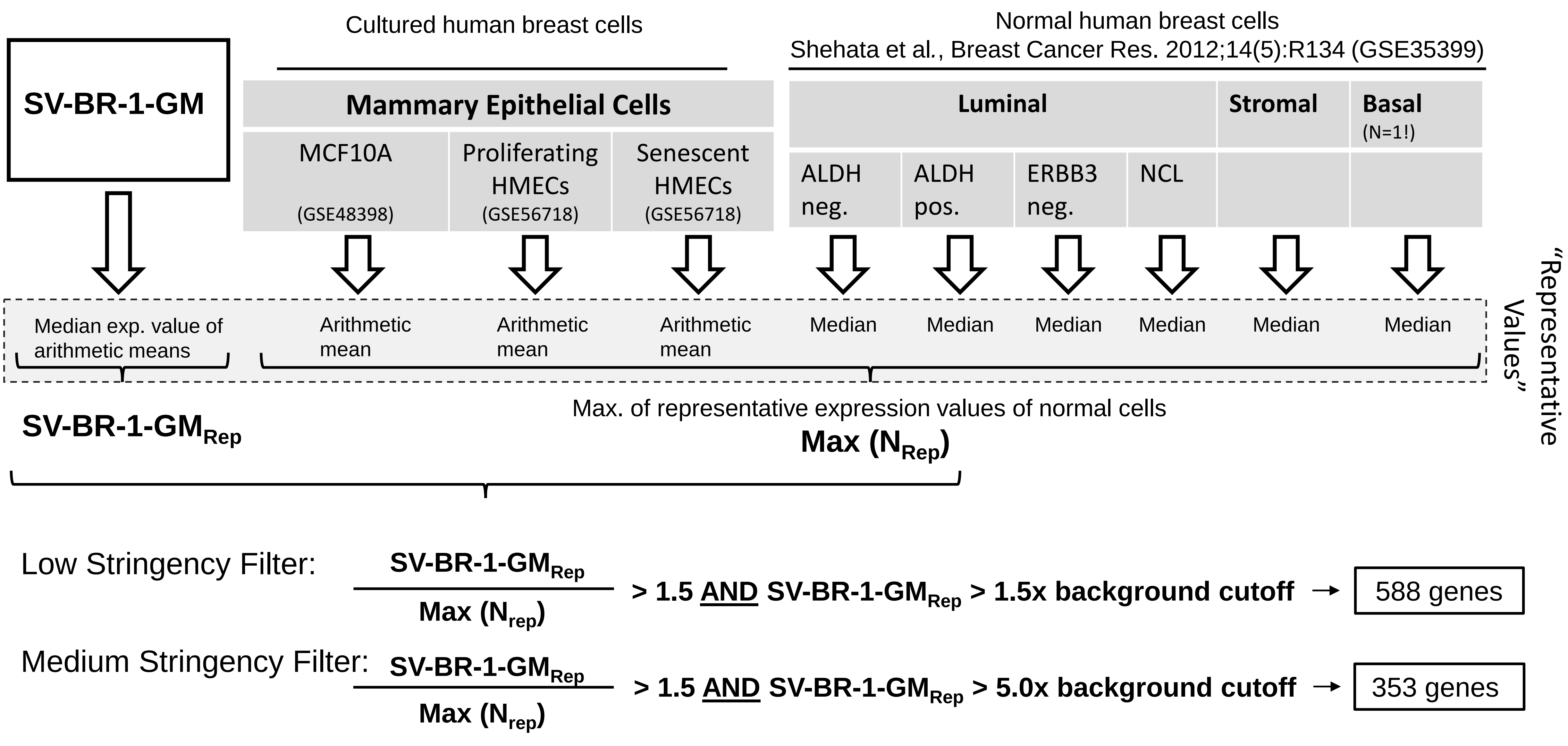

Low- and Medium-Stringency Filtration. See main text for details.

FIG. S8

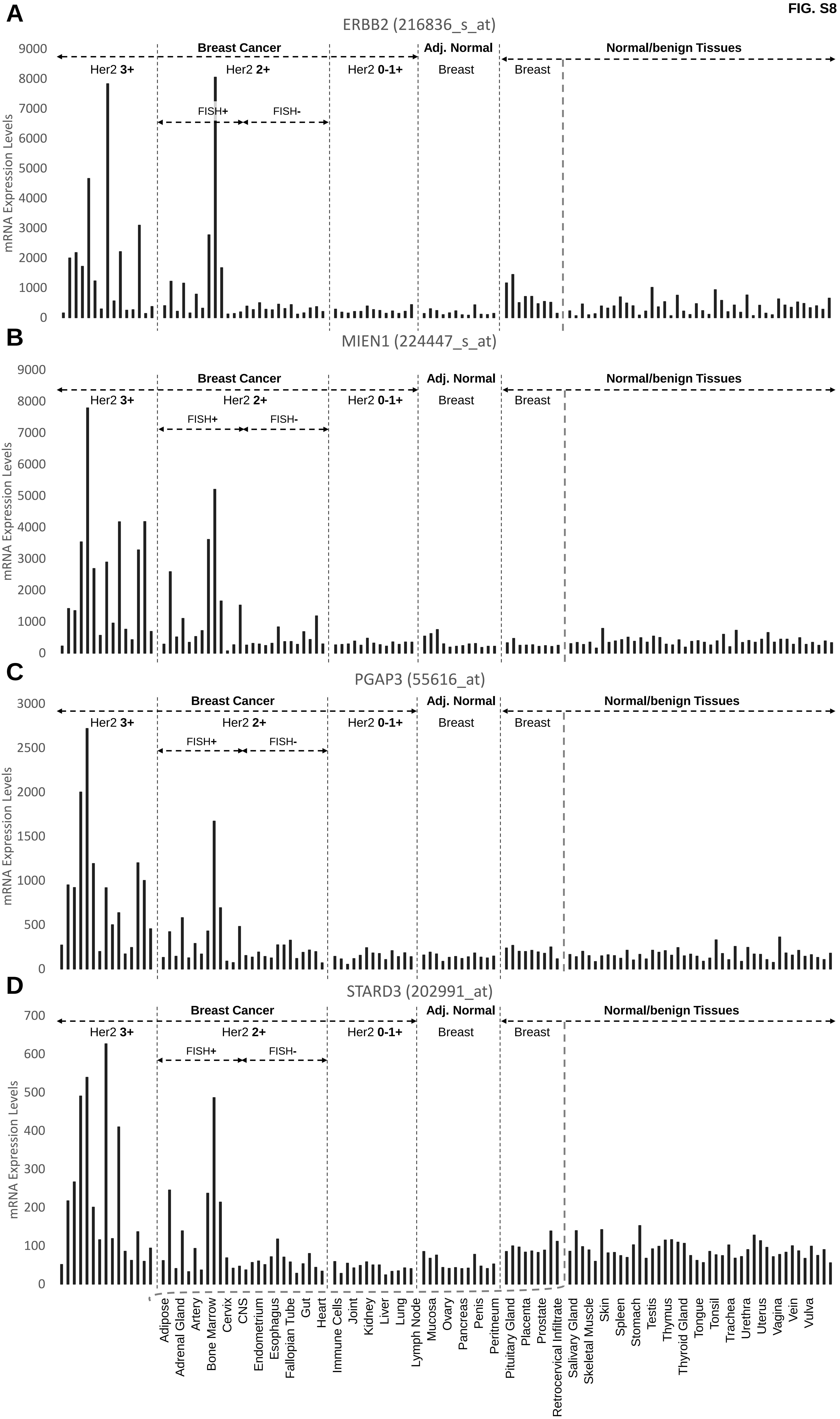
A
ERBB2 (216836_s_at)
B
MIEN1 (224447_s_at)
C
PGAP3 (55616_at)
D
STARD3 (202991_at)
mRNA Expression Levels
Breast Cancer
Adj. Normal
Normal/benign Tissues
Her2 3+
Her2 2+
Her2 0-1+
Breast
Breast
FISH+
FISH-
9000
8000
7000
6000
5000
4000
3000
2000
1000
0
3000
2500
2000
1500
1000
500
700
600
500
400
300
200
100
Adipose
Adrenal Gland
Artery
Bone Marrow
Cervix
CNS
Endometrium
Esophagus
Fallopian Tube
Gut
Heart
Immune Cells
Joint
Kidney
Liver
Lung
Lymph Node
Mucosa
Ovary
Pancreas
Penis
Peritneum
Pituitary Gland
Placenta
Prostate
Retrocervical Infiltrate
Salivary Gland
Skeletal Muscle
Skin
Spleen
Stomach
Testis
Thymus
Thyroid Gland
Tongue
Tonsil
Trachea
Urethra
Uterus
Vagina
Vein
Vulva

Legend to Figure S8

***In silico* screen for immunogen candidates. (A).** SV-BR-1-GM RNA samples were hybridized onto Illumina HumanHT-12 v4 Expression BeadChip arrays. SV-BR-1-GM expression data were compared to those of normal human breast cells provided in the Gene Expression Omnibus (GEO, NCBI) database as DataSets GSE35399 (Shehata et al., *Breast Cancer Res.* 2012;14(5):R134), GSE56718 (Lowe et al., *Genome Biol.* 2015 Sep 17;16:194), and GSE48398 (MCF10A only). Two serial filters (**Additional file 1: Figures S6 and S7**) were applied to the quantile-normalized expression values to enrich for genes likely differentiating SV-BR-1-GM from normal breast cells. Such genes represent candidate immunogens mediating SV-BR-1-GM's anti-cancer effect. After the (low stringency) first filter, 588 different genes were retained, of which after the (medium stringency) second filter, 353 remained. The latter genes were *in silico* verified on GEO DataSets GSE29431 (breast cancer tissues) and GSE7307 (nonmalignant tissues representing various organs; **Data Sheet 1 in Supplementary Material**). By means of this high stringency filtration/verification step, thirty-one genes were identified with expression levels higher in breast cancer than in a variety of nonmalignant tissues. Strikingly, among these thirty-one genes were four that mapped to 17q12 (**Table 4**), namely: **(A)** *ERBB2* (*HER2*/*neu*, Illumina probe 216836_s_at), **(B)** *MIEN1* (Illumina probe 224447_s_at), **(C)** *PGAP3* (Illumina probe 55616_at), and **(D)** *STARD3* (Illumina probe 202991_at).

A

Hypothetical Mechanism of Action of SV-BR-1-GM

FIG. S9

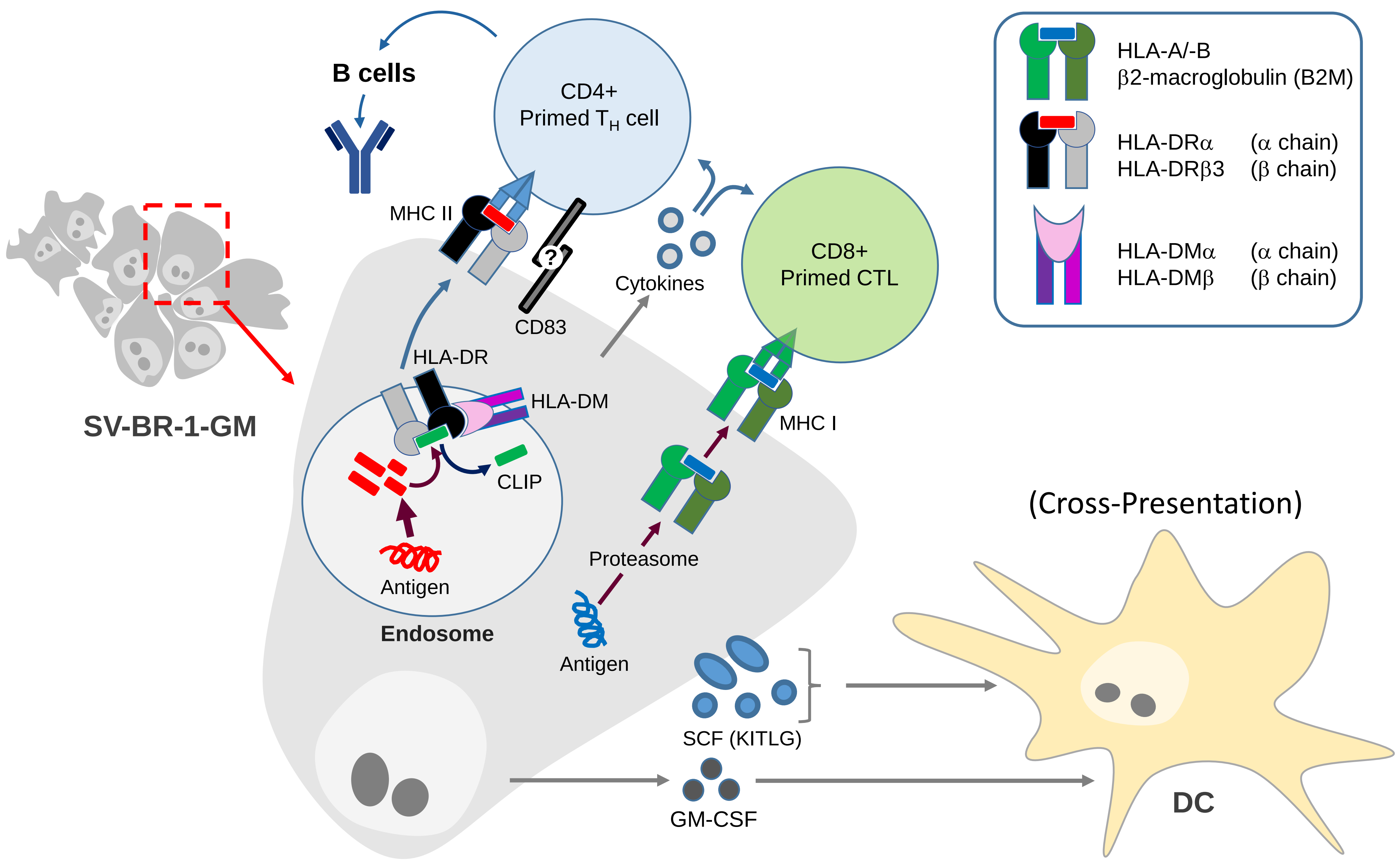

B

Hypothetical Mechanism of Action of SV-BR-1-GM

FIG. S9

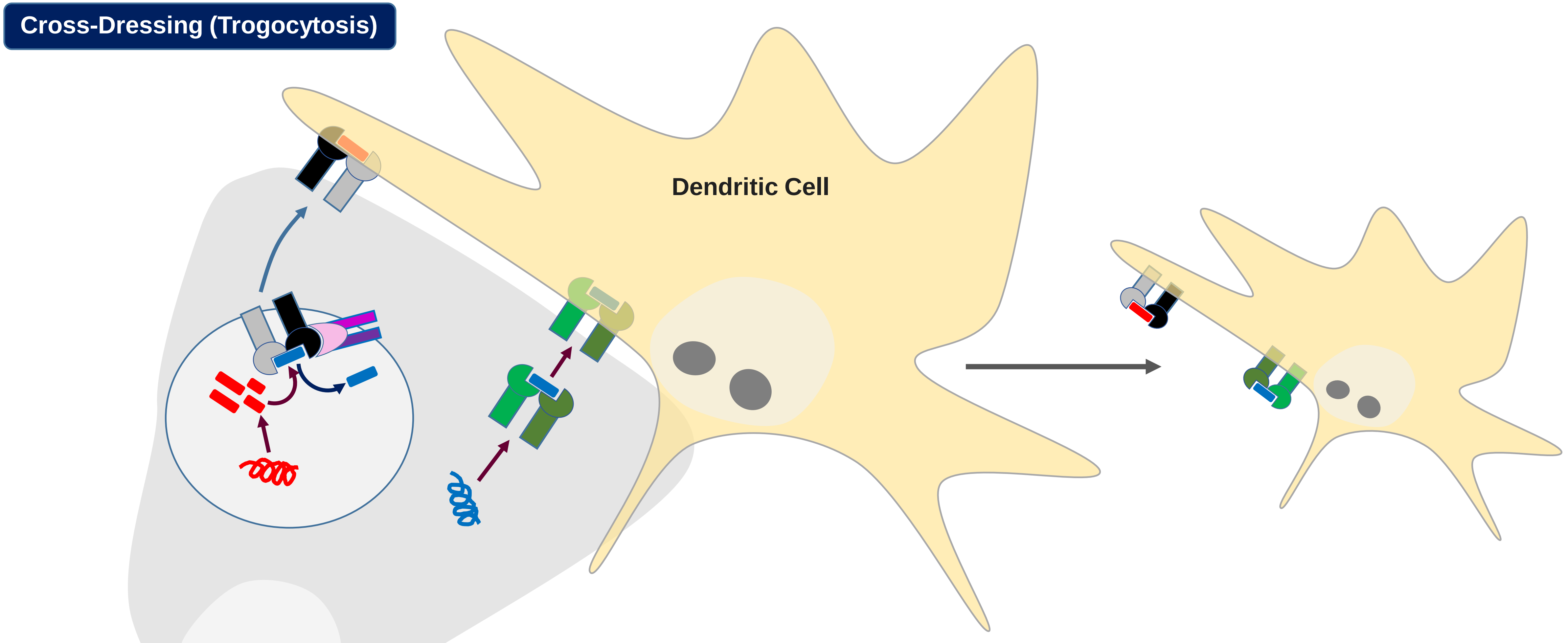

Cross-dressing of dendritic cells (DCs) with SV-BR-1-GM peptide-MHCs. Allogeneic SV-BR-1-GM cell surface MHCs (HLAs) loaded with SV-BR-1-GM antigens are directly transferred onto the cell surface of patient DCs by trogocytosis.

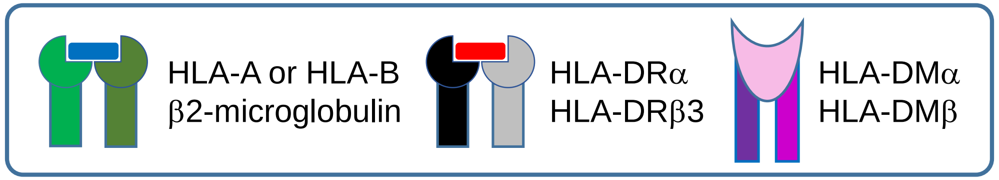

C

Hypothetical Mechanism of Action of SV-BR-1-GM

FIG. S9

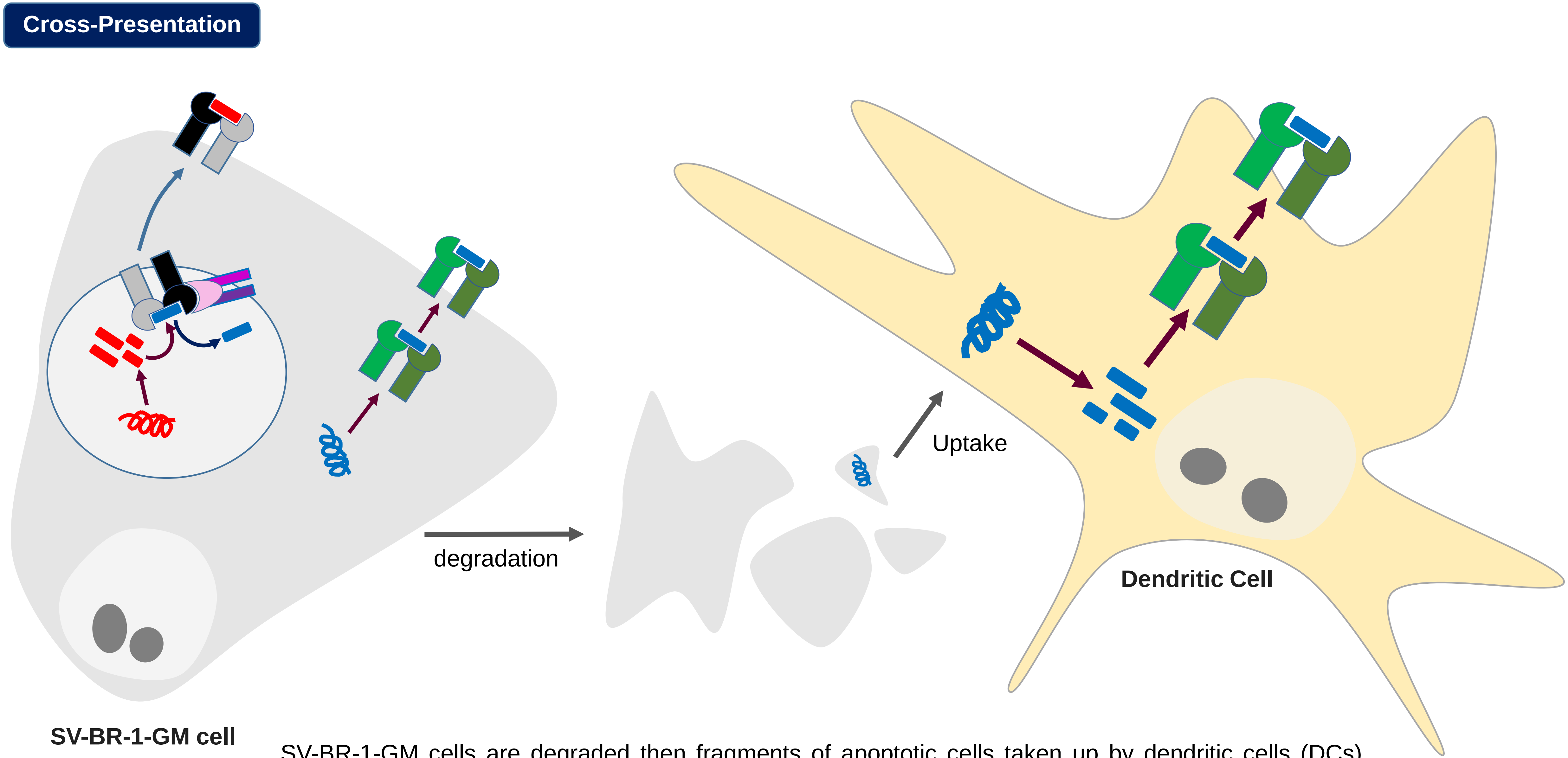

SV-BR-1-GM cells are degraded then fragments of apoptotic cells taken up by dendritic cells (DCs) from the patient. Inside DCs, SV-BR-1-GM antigens are proteolytically degraded then presented on cell surface MHCs (HLAs) to patient T cells (not shown).
