# Supplementary material for: SV-BR-1-GM, a Clinically Effective GM-CSF-Secreting Breast Cancer Cell Line, Expresses an Immune Signature and Directly Activates CD4+ T Lymphocytes
Source: Front Immunol. 2018 May 15;9:776. doi: 10.3389/fimmu.2018.00776 (PMC5962696; doi:10.3389/fimmu.2018.00776)
Supplement: Data Sheet 2 — Reagents and samples for quantitative RT-PCR and nCounter-based verification of gene expression are shown. [file Data_Sheet_2.docx]

**SV-BR-1-GM, a Clinically Effective GM-CSF-Secreting Breast Cancer Cell Line, Expresses an Immune Signature and Directly Activates CD4+ T Lymphocytes**

**Supplementary Data Sheet 2:**

**SUPPLEMENTARY MATERIALS AND METHODS**

Quantitative RT-PCR for validation of gene expression

**Table S1. Quantitative RT-PCR TaqMan® reagents.** Validation of gene expression on a subset of samples by quantitative RT-PCR was conducted on an ABI 7900HT real-time PCR instrument at the University of Minnesota Genomics Center using the commercially available TaqMan® assays (Thermo Fisher Scientific, Waltham, MA) listed.

| Gene Symbols | Assay Identifiers | Amplicon Lengths | Probes exon spanning? | RefSeqs (NCBI) |
| --- | --- | --- | --- | --- |
| CUL1 | Hs01117001_m1 | 113 | Yes | NM_003592.2 |
| HLA-DRA | Hs00219578_m1 | 129 | Yes | NM_019111.4 |
| HLA-DRB3 | Hs02339733_m1 | 74 | Yes | NM_022555.3 |
| HLA-DMA | Hs00185435_m1 | 100 | Yes | NM_006120.3 |
| HLA-DMB | Hs00157943_m1 | 148 | Yes | NM_002118.4 |
| CD74 | Hs00269961_m1 | 102 | Yes | NM_001025158.2 NM_001025159.2  NM_004355.3 |

**Table S2. Samples for quantitative RT-PCR.** Validation of gene expression on a subset of samples by quantitative RT-PCR was conducted at the University of Minnesota Genomics Center (MN) using the samples listed. RIN, RNA Integrity Number; OD, Optical Density.

| Samples | RIN | [RNA] [ng/μl] | OD 260/280 | OD 260/230 |
| --- | --- | --- | --- | --- |
| MCB cryo | 7.5 | 587.1 | 1.96 | 1.97 |
| CP Lot IV cryo | 6.9 | 935.1 | 2.06 | 1.9 |
| CP Lot VIII cryo | 10 | 108.1 | 1.84 | 2.23 |
| CP Lot VIII cryo | 9.9 | 107.7 | 1.89 | 1.91 |
| CP Lot IV culture | 9.9 | 514.4 | 2 | 1.91 |
| CP Lot VIII culture | 10 | 71.24 | 1.8 | 2.41 |

Gene expression by nCounter

**Table S3. nCounter CodeSets.**

| **Gene Symbol (NCBI)** | **CodeSet Sequence** | **Isoforms Hit By Probe / Comments** | **Isoforms Not Hit By Probe** |
| --- | --- | --- | --- |
| **ADA** | TCCAAGAAGACCATGATCTCAATAGTCAGTTACTGATGCTCCTGAACCCTATGTGTCCATTTCTGCACACACGTATACCTCGGCATGGCCGCGTCACTTC | NM_001322050.1;NM_001322051.1;NM_000022.3;NR_136160.1 |  |
| **ADGRE5** | GAACCTGCATTCCAAGAAGCAAGCCGAACTGGAGGAGATATATGAAAGCAGCATCCGTGGTGTCCAACTCAGACGCCTCTCTGCCGTCAACTCCATCTTT | NM_001784.4;NM_078481.3;NM_001025160.2;XM_017027547.1;XM_011528451.2 |  |
| **ADRM1** | AAGTGGACCTGGCCAGTGTGCTGACGCCGGAGATAATGGCTCCCATCCTCGCCAACGCGGATGTCCAGGAGCGCCTGCTTCCCTACTTGCCATCTGGGGA | NM_001281438.1;XM_017027610.1;NM_001281437.1;XM_017027607.1;XM_005260257.1;XM_017027608.1;NM_007002.3;XM_011528503.1;XM_017027605.1;XM_017027602.1;XM_017027609.1;XM_017027603.1;NM_175573.2;XM_017027604.1;XM_017027606.1 |  |
| **APTX** | CCCAAAATGCAGGTTTACAAAGATGAGCAGGTGGTGGTGATAAAGGATAAATACCCAAAGGCCCGTTACCATTGGCTGGTCTTACCGTGGACCTCCATTT | XM_017014833.1;XM_011517939.2;XM_017014834.1;XR_929276.2;XM_006716791.3;NR_036576.1;NM_001195248.1;XR_929277.2;NM_001195254.1;XM_017014837.1;XR_001746325.1;NM_175073.2;NR_036578.1;XM_017014838.1;XM_006716792.2;XR_001746324.1;NM_175069.2;XM_017014835.1;XM_017014832.1;XM_017014831.1;XM_017014836.1;NR_036579.1;NM_001195251.1;XM_011517938.1;XR_001746326.1;NR_036577.1;NM_001195250.1;NM_001195249.1;NM_001195252.1 | XR_929279.1;XR_428423.2 |
| **B2M** | CGGGCATTCCTGAAGCTGACAGCATTCGGGCCGAGATGTCTCGCTCCGTGGCCTTAGCTGTGCTCGCGCTACTCTCTCTTTCTGGCCTGGAGGCTATCCA | NM_004048.2;XM_005254549.3 |  |
| **CAV1** | AACCGCGACCCTAAACACCTCAACGATGACGTGGTCAAGATTGACTTTGAAGATGTGATTGCAGAACCAGAAGGGACACACAGTTTTGACGGCATTTGGA | NM_001172897.1;NM_001172896.1;NM_001172895.1;NM_001753.4 |  |
| **CD58** | GTGCTTGAGTCTCTTCCATCTCCCACACTAACTTGTGCATTGACTAATGGAAGCATTGAAGTCCAATGCATGATACCAGAGCATTACAACAGCCATCGAG | NM_001779.2;XM_017002869.1;NM_001144822.1;NR_026665.1 |  |
| **CD74** | TTCAGCCCCCAGCCCCTCCCCCATCTCCCACCCTGTACCTCATCCCATGAGACCCTGGTGCCTGGCTCTTTCGTCACCCTTGGACAAGACAAACCAAGTC | NM_001025159.2;NM_004355.3;XM_017010089.1;XM_017010090.1;NM_001025158.2 |  |
| **CD83** | CTGTTCTTGAAGCAGTAGCCTAACACACTCCAAGATATGGACACACGGGAGCCGCTGGCAGAAGGGACTTCACGAAGTGTTGCATGGATGTTTTAGCCAT | NM_001040280.1;NM_001251901.1;NM_004233.3 |  |
| **CSF2** | AGATGAGGCTGGCCAAGCCGGGGAGCTGCTCTCTCATGAAACAAGAGCTAGAAACTCAGGATGGTCATCTTGGAGGGACCAAGGGGTGGGCCACAGCCAT | NM_000758.3 |  |
| **CXCL16** | CCATGGGTTCAGGAATTGATGAGCTGTCTTGATCTCAAAGAATGTGGACATGCTTACTCGGGGATTGTGGCCCACCAGAAGCATTTACTTCCTACCAGCC | NM_001100812.1;NM_022059.3 |  |
| **CXCL8** | ACAGCAGAGCACACAAGCTTCTAGGACAAGAGCCAGGAAGAAACCACCGGAAGGAACCATCTCACTGTGTGTAAACATGACTTCCAAGCTGGCCGTGGCT | NM_000584.3 |  |
| **DGUOK** | TTGTAAAGAATCTGTAACCAATACCATGAAGTTCAGGCTGTGATCTGGGCTCCCTGACTTTCTGAAGCTAGAAAAATGTTGTGTCTCCCAACCACCTTTC | NR_134896.1;NM_080916.2;NR_134898.1;NM_001318863.1;NM_080918.2;NM_001318859.1;NR_134894.1;NR_134897.1;NM_001318861.1;XM_011532647.2;XR_244926.3;NM_001318860.1;NR_134893.1;NR_134895.1;XR_001738656.1;NM_001318862.1 |  |
| **GNG5** | ACGACCCACCGACCCACGAATCGGCCCGGCCGTCGCGTGCACCATGTCTGGCTCCTCCAGCGTCGCCGCTATGAAGAAAGTGGTTCAACAGCTCCGGCTG | NM_005274.2 |  |
| **HLA-A** | AGGAGGGGCCGGAGTATTGGGACCAGGAGACACGGAATGTGAAGGCCCAGTCACAGACTGACCGAGTGGACCTGGGGACCCTGCGCGGCTACTACAACCA | Targets HLA-A*11:01 | NM_002116.7;NM_001242758.1;XR_430999.1;XM_005275331.2;XM_017030288.1 |
| **HLA-A** | AGGAGGGGCCGGAGTATTGGGACGAGGAGACAGGGAAAGTGAAGGCCCACTCACAGACTGACCGAGAGAACCTGCGGATCGCGCTCCGCTACTACAACCA | Targets HLA-A*24:02 | NM_002116.7;NM_001242758.1;XR_430999.1;XM_005275331.2;XM_017030288.1 |
| **HLA-B** | AGCCTGCGGAACCTGCGCGGCTACTACAACCAGAGCGAGGCCGGGTCTCACATCATCCAGAGGATGTATGGCTGCGACCTGGGGCCCGACGGGCGCCTCC | Targets HLA-B*35:08; also targets HLA-A/C/E RefSeq transcripts at >91%. | XR_926175.1;XM_011514557.1;NM_005514.7 |
| **HLA-B** | AGCCTGCGGAACCTGCGCGGCTACTACAACCAGAGCGAGGCCGGGTCTCACACTTGGCAGACGATGTATGGCTGCGACCTGGGGCCGGACGGGCGCCTCC | Targets HLA-B*55:01; also targets HLA-A/C RefSeq transcripts at >90%. | XR_926175.1;XM_011514557.1;NM_005514.7 |
| **HLA-DMA** | TTATTTGACAAAGAGTTCTGCGAGTGGATGATCCAGCAAATAGGGCCAAAACTTGATGGGAAAATCCCGGTGTCCAGAGGGTTTCCTATCGCTGAAGTGT | NM_006120.3 |  |
| **HLA-DMB** | CCCGTGAGCTGGAAGGAACAGATTTAATATCTAGGGGCTGGGTATCCCCACATCACTCATTTGGGGGGTCAAGGGACCCGGGCAATATAGTATTCTGCTC | NM_002118.4 |  |
| **HLA-DRA** | GGCCAACATAGCTGTGGACAAAGCCAACCTGGAAATCATGACAAAGCGCTCCAACTATACTCCGATCACCAATGTACCTCCAGAGGTAACTGTGCTCACG | NM_019111.4 |  |
| **HLA-DRB1** | CCTATAACTTGGAATGTGGGTGGAGGGGTTCATAGTTCTCCCTGAGTGAGACTTGCCTGCTTCTCTGGCCCCTGGTCCTGTCCTGTTCTCCAGCATGGTG | NM_001243965.1;NM_002124.3; also targets HLA-DRB5 (XM_011514562) at 95%. | XM_011547738.2 |
| **HLA-DRB3** | TTGGAGCTGCGTAAGTCTGAGTGTCATTTCTTCAATGGGACGGAGCGGGTGCGGTACCTGGACAGATACTTCCATAACCAGGAGGAGTTCCTGCGCTTCG | Targets HLA-DRB3*01:01; also targets HLA-DRB1 RefSeq transcripts at >90%. | NM_022555.3 |
| **HLA-DRB3** | TTGGAGCTGCTTAAGTCTGAGTGTCATTTCTTCAATGGGACGGAGCGGGTGCGGTTCCTGGAGAGACACTTCCATAACCAGGAGGAGTACGCGCGCTTCG | Targets HLA-DRB3*02:02; also targets HLA-DRB1 RefSeq transcripts at >90%. | NM_022555.3 |
| **HLA-DRB6** | TTCCAGGCAGTTACGGAACTGGGGCGGCCTGTCGCAGAGAACTGGAACAGCCAGAAGGGCATCCCGGAGGAGAAGCGGGACAAGATGGACGACTACTGCA | NR_001298.1 |  |
| **HLA-F** | TGCGCTCCTGGACCGCGGCGGACACCGTGGCTCAGATCACCCAGCGCTTCTATGAGGCAGAGGAATATGCAGAGGAGTTCAGGACCTACCTGGAGGGCGA | XR_001743376.1;XR_001743374.1;XM_017010814.1;XM_017010815.1;NM_001098478.1;XM_011514564.1;NM_018950.2;XM_017010811.1;XM_017010813.1;NM_001098479.1;XM_017010810.1;XR_001743373.1;XM_017010812.1 |  |
| **ICAM3** | AGCGTCCAGCTGCGAGTCCTGTATGGTCCCAAAATTGACCGAGCCACATGCCCCCAGCACTTGAAATGGAAAGATAAAACGAGACACGTCCTGCAGTGCC | NM_001320608.1;NM_001320606.1;NM_001320605.1;NM_002162.4 |  |
| **IL10** | AAGGATCAGCTGGACAACTTGTTGTTAAAGGAGTCCTTGCTGGAGGACTTTAAGGGTTACCTGGGTTGCCAAGCCTTGTCTGAGATGATCCAGTTTTACC | NM_000572.2 |  |
| **IL15** | AGGGTGATAGTCAAATTATGTATTGGTGGGGCTGGGTACCAATGCTGCAGGTCAACAGCTATGCTGGTAGGCTCCTGCCAGTGTGGAACCACTGACTACT | NM_172175.2;NM_000585.4;NR_037840.2 |  |
| **IL18** | GACAGTCAGCAAGGAATTGTCTCCCAGTGCATTTTGCCCTCCTGGCTGCCAACTCTGGCTGCTAAAGCGGCTGCCACCTGCTGCAGTCTACACAGCTTCG | XM_011542805.1;NM_001562.3;XM_011542806.2;NM_001243211.1 |  |
| **IL6** | GGCACTGGCAGAAAACAACCTGAACCTTCCAAAGATGGCTGAAAAAGATGGATGCTTCCAATCTGGATTCAATGAGGAGACTTGCCTGGTGAAAATCATC | XM_011515390.2;NM_001318095.1;XM_005249745.4;NM_000600.4 |  |
| **KITLG** | AGGACTCTATTTTAAGGACTGCGGGACTTGGGTCTCATTTAGAACTTGCAGCTGATGTTGGAAGAGAAAGCACGTGTCTCAGACTGCATGTACCATTTGC | NM_003994.5;NM_000899.4 |  |
| **PSMA4** | GTACATTGGCTGGGATAAGCACTATGGCTTTCAGCTCTATCAGAGTGACCCTAGTGGAAATTACGGGGGATGGAAGGCCACATGCATTGGAAATAATAGC | NM_001330676.1;NM_001102668.2;NM_001330673.1;NM_002789.5;NM_001102667.2;NM_001330675.1 |  |
| **RPL38** | TCACAGCCCGACGAAAGGATGCCAAATCTGTCAAGATCAAGAAAAATAAGGACAACGTGAAGTTTAAAGTTCGATGCAGCAGATACCTTTACACCCTGGT | NM_001035258.1;NM_000999.3 |  |
| **TMEM14C** | AGGTTCTACCACTCTGGAAAATTCATGCCTGCAGGTTTAATTGCAGGTGCCAGTTTGCTGATGGTCGCCAAAGTTGGAGTTAGTATGTTCAACAGACCCC | NM_001165258.1;NM_016462.3 |  |
| **TNFSF14** | GGCGTGTCAGCCCTGCTCCAGACACCTTGGGCATGGAGGAGAGTGTCGTACGGCCCTCAGTGTTTGTGGTGGATGGACAGACCGACATCCCATTCACGAG | XM_017027418.1;XM_017027417.1;NM_003807.4;XR_001753777.1;XR_936212.2;NM_172014.3 |  |
| **UBE3C** | TAGAGAGTAGATGTTCAAGAAAGAGTGGTGGAGCACCCTGGCTTTTCTATTTCGTTTTAACTGTTGGCGAAAATTATTTGGGGGCCCTCTCTGAGGAAGG | XR_927552.2;NM_014671.2;XM_017012818.1;XM_005249564.4 |  |
